# Surveillance of hepatocellular carcinoma (HCC) patients using Protein Induced by Vitamin K (PIVKA-II): A cost-utility analysis for Hong Kong

**DOI:** 10.1371/journal.pone.0353882

**Published:** 2026-07-17

**Authors:** Man-Fung Yuen, David Wastlund, Tammie Tan Yan Lin, Qishi Zheng, Junqiao Chen, Kwan-Lung Ko

**Affiliations:** 1 Department of Medicine, School of Clinical Medicine & State Key Laboratory of Liver Research, The University of Hong Kong and Queen Mary Hospital, Hong Kong, China; 2 Vista Health Private Limited, Singapore, Singapore; 3 Roche Diagnostics (Asia Pacific) Limited, Singapore, Singapore; 4 Roche Hong Kong Limited, Hong Kong SAR, China; Kaohsiung Medical University Hospital, TAIWAN

## Abstract

**Background & Aims:**

Hepatocellular carcinoma (HCC) is a leading cause of cancer-related death in Hong Kong. Common risk factors include chronic hepatitis B (CHB) infection and/ or liver cirrhosis (LC). Routine HCC surveillance using ultrasound plus alpha-fetoprotein (AFP) for patients with these conditions is recommended, but many patients still face limited access to ultrasound. For these, biomarker-based surveillance using a combination of protein induced by vitamin K absence or antagonist-II (PIVKA-II) plus AFP could provide a valuable alternative.

**Methods:**

This study compared the cost-utility of routine HCC surveillance using ‘PIVKA-II + AFP’ to the recommended standard-of-care in Hong Kong: surveillance using ‘US + AFP’. A health economic model using a Markov-microsimulation framework was adapted using published local data and settings. The model was used to compare diagnostic outcomes, overall costs, quality-adjusted life years (QALYs), and cost-utility of the alternative HCC surveillance methods.

**Results:**

Compared to the current recommended standard-of-care, the results suggested that HCC surveillance using ‘PIVKA-II + AFP’ could improve detection of HCC during early-stage disease by 4.6 percentage points, thus enabling access to more effective treatment options for some patients. It could also reduce false positive diagnoses by 7.2%, which could alleviate the need for costly confirmatory testing. Overall, PIVKA-II + AFP’ was associated with a gain of 0.019 QALYs and cost savings of HK $ 4,144 per individual. The results were particularly favorable for non-cirrhotic CHB patients.

**Conclusions:**

HCC surveillance using ‘PIVKA-II + AFP’ could be a feasible option for patients with CHB and/ or LC who face limited access to ultrasound-based surveillance. The combination has the potential to improve early-stage HCC detection, facilitating timely intervention and improving both survival outcomes and patient quality of life.

## Introduction

Hepatocellular carcinoma (HCC) is one of the most common forms of cancer, accounting for over 80% of primary liver cancers worldwide. HCC also imposes a substantial disease burden and ranks among the leading causes of cancer-related deaths, estimated to be the fourth most common cause of cancer-related death worldwide [1,2]. HCC typically develops in patients with cirrhosis of the liver, which itself results from causes such as alcoholic liver disease (ALD) or metabolic dysfunction–associated steatotic liver disease (MASLD) or following infection with hepatitis B or C viruses (HBVs/ HCVs) [1]. Hong Kong, a city in a high-endemic region of liver diseases, observes disproportional mortality related to HCC, with age-standardized incidence rates of 18.9 for males & 5.1 for females per 100,000 standard population in 2021 [3].

Effective and potentially curative treatments exist, particularly where HCC is detected during its early stages, e.g., surgical resection, local ablation, or liver transplantation. However, symptoms attributable to HCC are usually absent in early stages, and many patients are therefore diagnosed with advanced disease (BCLC stages B/C/D [4]), often precluding potentially curative therapies [5,6]. This has resulted, in part, in a 5-year overall survival rate of 12% and median overall survival (OS) following diagnosis ranging from 6 to 20 months [7]. By contrast, in healthcare systems with higher early-stage detection rates, e.g., Taiwan and Japan, median OS may exceed 60 months [8]. To improve the chances of earlier detection and associated survival, both domestic and international guidelines strongly recommend including high-risk patients in biannual surveillance [5,9–11].

The combination of ultrasound (US) and α-fetoprotein (AFP) is recommended as the standard of care for HCC surveillance in Hong Kong clinical practice [5]. The addition of clinical biomarkers to US has been suggested to improve the sensitivity for HCC [12], particularly for early-stage tumors [13]. AFP is an oncofetal glycoprotein that can signal HCC growth and may improve the clinical performance of HCC surveillance when used in combination with US or other biomarkers [9,14]. However, ‘US + AFP’ has some drawbacks. First, assessment using ultrasound is subjective, and multiple factors may impact the accuracy of US assessment, including the quality of equipment, experience of the sonographer, and presence of hepatic steatosis [15]. Second, one downside of the increased sensitivity for early-stage HCC when combining US with AFP is its decrease in specificity, since increased AFP serum levels can also occur within patients without HCC [13]. This could lead to more false positive diagnoses requiring unwarranted confirmatory testing for HCC. Third, capacity constraints for ultrasound assessment are said to remain a critical bottleneck to ensure widespread, effective, sustainable, and equitable access for patients at risk for HCC. For patients with limited access to ultrasound, biomarker-based methods for HCC surveillance could constitute a valuable alternative, with an increasing body of evidence and clinical consensus supporting their usage [16,17].

New diagnostic strategies and tools for the early detection of HCC represent one of the most promising approaches to reducing the growing burden of the disease [18]. Emerging tools for the detection of HCC include the biomarker protein induced by vitamin K absence or antagonist-II (PIVKA-II; also known as des-γ-carboxy prothrombin [DCP]). PIVKA-II is an immature form of prothrombin that abnormally accumulates because of the deficiency of γ-glutamyl carboxylase in HCC cells [16]. Recent studies also describe PIVKA-II as an autologous growth factor to stimulate HCC growth, and an independent predictor of microvascular invasion in HCC [19]. As such, PIVKA-II has been increasingly studied and applied for its ability to increase the diagnostic performance of HCC surveillance strategies, and combining PIVKA-II with AFP may further increase the diagnostic performance [16,20]. Expert have highlighted the need for more evidence on the cost-effectiveness of screening with PIVKA-II plus AFP to guide the HCC surveillance policy in Hong Kong [17]. Consequently, the objective of this study is to evaluate the cost-utility of surveillance using PIVKA-II plus AFP versus recommended standard-of-care to detect HCC in patients with cirrhosis or chronic hepatitis B infection in Hong Kong.

## Methods

A health economic state-transition microsimulation model in Microsoft Excel was developed to simulate health and cost outcomes under alternative surveillance strategies. The model has previously been described elsewhere [21], and was critically appraised and adapted to reflect Hong Kong clinical settings.

### Study population

In line with the Hong Kong clinical guidelines [22], the model was adapted to focus on two target populations: non-cirrhotic chronic hepatitis B (CHB) patients and liver cirrhosis (LC) patients. For the base case, a mixed population was assumed (CHB: 85% - LC: 15%), but scenarios with surveillance targeting only LC or CHB patients were also explored. Patient starting age at surveillance differed by etiology, ranging between 45 and 56 years old (Table 1), based on the mean age for various etiologies reported in a local study [23]. The same study reported the etiologies of LC patients to be: hepatitis B (91.5%), hepatitis C infection (5.2%), ALD (1.8%), and non-alcoholic fatty liver disease (NAFLD) (1.6%) [23]. Unless HCC was detected, routine surveillance was assumed to stop once patients turned 70 years old.

**Table 1:** Model data inputs for key parameters.

Name	Value	Sources & notes
Population and epidemiology		
Age of surveillance		
CHB patient	45–75 years	Fung, et al. 2007 [23]
LC, due to ALD	56 - 75 years	
LC, due to HBV	45 - 75 years	
LC, due to HCV	56 - 75 years	
LC, due to NAFLD	53 - 75 years	
Incidence of HCC		
HCC, incidence rate annual if CHB	0.31%	Wong, et al. 2018 [24]
HCC, incidence rate annual if ALD	0.47%	
HCC, incidence rate annual if HBV	2.19%	
HCC, incidence rate annual if HCV	0.35%	
HCC, incidence rate annual if NAFLD	1.09%	
Incidence of DCLC		
DCLC, incidence rate annual if ALD	7.3%	NICE guideline NG50 [25], Fleming 2010 [26]
DCLC, incidence rate annual if HBV	5.0%	NICE guideline NG50 [25], Dakin 2010 [27]
DCLC, incidence rate annual if HCV	4.0%	NICE guideline NG50 [25], Wright 2006 [28]
DCLC, incidence rate annual if NAFLD	3.8%	Chris Estes 2017 [29]
Diagnostic accuracy		
US + AFP (for all population)		
Sensitivity, US + AFP, early	63.0%	Tzartzeva K, et al. 2018 [13]
Sensitivity, US + AFP, all	97.0%	
Specificity, US + AFP, all	84.0%	
PIVKA-II + AFP (LC population)		
Sensitivity, PIVKA-II + AFP, early	75.6%	Pinjaroen et al. 2026 [30]
Sensitivity, PIVKA-II + AFP, all	86.8%	
Specificity, PIVKA-II + AFP, all	75.9%	
PIVKA-II + AFP (CHB population)		
Sensitivity, PIVKA-II + AFP, early	82.1%	Pham et al. (2025) [31]
Sensitivity, PIVKA-II + AFP, all	84.4%	
Specificity, PIVKA-II + AFP, all	96.0%	
Ultrasound alone (for all population)		
Sensitivity, US, early	45.0%	Tzartzeva K, et al. 2018 [13]
Sensitivity, US, all	78.0%	
Specificity, US, all	92.0%	
HCC treatment		
HCC treatment distribution for EARLY detected		
OLT	22.5%	Yau et al. 2014 [32]
Resection	15.7%	
RFA	34.8%	
TACE	25.8%	
Systemic Treatment	0.0%	
BSC	1.1%	
HCC treatment distribution for LATE detected		
OLT	6.1%	Yau et al. 2014 [32]
Resection	3.1%	
RFA	6.8%	
TACE	30.8%	
Systemic Treatment	1.7%	
BSC	51.6%	
Utilities		
CHB	0.773	Zhang et al. 2021 [33]
LC	0.750	Zhang et al. 2021 [33]
DCLC	0.683	Zhang et al. 2021 [33]
HCC	0.640	Zhang et al. 2021 [33]
BSC & palliative	0.615	Zhang et al. 2021 [33]
Surveillance costs		
US + AFP	HK$690.88	Yuen et al. 2000 [34], adjusted for inflation
PIVKA-II + AFP	HK$224.06	Roche diagnostics, data on file, (Appendix 1)
US	HK$552.71	Yuen et al. 2000 [34], adjusted for inflation
Event costs		
LC, annual	HK$20,500	Local costing data on file, verified by KOL [35]
DCLC, annual	HK$116,248	
True positive for HCC (confirmatory)	HK$14,018	
False positive for HCC	HK$10,228	
Symptomatic diagnosis	HK$5,296	
Follow-up after HCC (biannual CT scan)	HK$5,296	
Treatment costs		
OLT, per operation	HK$1,206,152	Local costing data on file, verified by KOL [35]
Post-OLT follow up (year 1)	HK$712,601	
Post-OLT follow up (year 2+)	HK$112,896	
Resection	HK$121,760	
RFA	HK$56,430	
TACE	HK$56,487	
Systemic treatment, annual	HK$17,586	
BSC, per month	HK$51,574	

Abbreviations: LC = Liver cirrhosis; DCLC = Decompensated liver cirrhosis; HCC 0/A = Hepatocellular carcinoma – Barcelona Liver Clinic Stages 0 and A; HCC B/C/D = Hepatocellular carcinoma – Barcelona Liver Clinic Stages B, C and D; WL = Waiting list for liver transplantation; OLT = Orthotopic liver transplantation; RFA = Radiofrequency thermal ablation; TACE = Transarterial chemoembolization; PEI = Punctual ethanol injection; BSC = Best supportive care; SYS = Systemic Therapy.

### Intervention and comparators

This study sought to compare bi-annual surveillance using ‘PIVKA-II + AFP’ to the current recommended standard-of-care in Hong Kong: ‘US + AFP’. To further evaluate the impact of these biomarkers on HCC surveillance, US was also evaluated as standalone surveillance method.

### Outcomes and analytical settings

A cost-utility analysis was conducted based upon 50,000 microsimulations. Diagnostic outcomes included the share of HCC cases detected during early-stage, as well as the total number of true positive, false negative, and false positive diagnoses. The primary cost-effectiveness outcomes were the total cost of direct healthcare services, total quality-adjusted life years (QALYs), and cost per QALY gained. For sensitivity analysis, the net monetary benefit (NMB) was used to assess the impact on cost-effectiveness.

Costs of direct healthcare services, including surveillance, confirmatory diagnostics, treatments, and prognosis, were extracted from local publications (Table 1) [36]. All costs are reported in HK$ (HK$1 = US$0.13); costs were inflated to the value of the year 2024 using CPI inflation rate [37]. A health system payer perspective with a lifetime horizon was adopted to simulate the lifetime cost and benefits for various surveillance strategies, at an annual discount rate of 3.0% (commonly used in cost-effectiveness studies in Hong Kong) [38,39]. In the absence of an official threshold for cost-effectiveness in Hong Kong, a target threshold of HK$ 422,242 per QALY gained (i.e., the GDP per capita in Hong Kong in 2024 [40]) was used. Reporting was based upon the Consolidated Health Economic Evaluation Reporting Standards (CHEERS) checklist [41].

### Simulation model structure and treatment pathways

**Fig 1:**
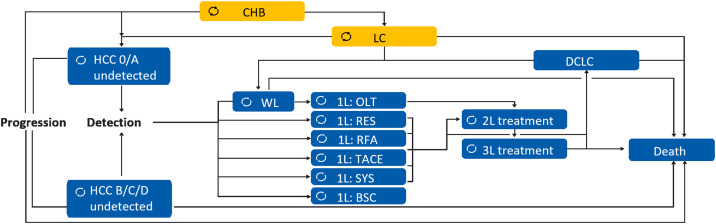
Overview of health states and model schematics. Abbreviations: CHB: Chronic hepatitis B; LC = Liver cirrhosis; DCLC = Decompensated liver cirrhosis; HCC 0/A = Hepatocellular carcinoma – Barcelona Liver Clinic Stages 0 and A; HCC B/C/D = Hepatocellular carcinoma – Barcelona Liver Clinic Stages B, C and D; WL = Waiting list for liver transplantation; OLT = Orthotopic liver transplantation; RES = resection; RFA = Radiofrequency thermal ablation; TACE = Transarterial chemoembolization; BSC = Best supportive care; SYS = Systemic therapy.

A microsimulation framework was used based upon 6-month cycles. An overview of the model’s states is shown in Fig 1.This framework has previously been validated and is consistent with most previous cost-effectiveness analyses of HCC surveillance [42]. For every model cycle, patients could remain in the current model state, or transition to another state, subject to conditional probabilities. Results for HCC surveillance uptake, surveillance outcome, disease progression, treatment, and survival status were simulated for each cycle and patient. Total costs and QALYs/life years for each patient were calculated based on the time spent within each model state. The accuracy of the disease progression and treatment pathways for Hong Kong was verified by local clinical experts.

Patients entered the model with either CHB or LC. From these stages, progression to decompensated liver cirrhosis (DCLC), early-stage HCC (HCC 0/A), late-stage HCC (B/C/D), or death was possible. For each model cycle, all HCC-positive individuals could be detected or undetected, depending on the accuracy of the surveillance method. Non-HCC individuals could also be correctly diagnosed, or suspected of HCC (false positive test), which would require confirmatory testing using computed tomography (CT) scan. To compare the outcomes between surveillance methods, each individual’s health journey was simulated 4 times, one for each intervention compared.

For individuals with confirmed HCC, 6 treatments were possible in the model: orthotopic liver transplantation (OLT), resection, Radiofrequency thermal ablation (RFA), Transarterial Chemoembolization (TACE), Systemic therapy (Sorafenib), or best supportive care (BSC). The probability of receiving each treatment depended on the stage of HCC when diagnosed (early- vs late-stage). Each treatment was associated with 1) a survival curve, 2) an average treatment cost, and 3) a quality-of-life value. The survival of undetected HCC-cases was based upon natural history for HCC patients by early- vs late-stage HCC. For patients treated with OLT, an average waiting time of 6 months prior to the transplantation was assumed.

### Model parameters

Data for the analysis were identified using a targeted literature review and validated by Hong Kong clinical experts. Data from Hong Kong were prioritized, followed by data from other territories in the Asia-Pacific region. Where available, data from the meta-analysis were preferred over other sources. A list of key model parameters for the base case analysis can be found in Table 1. More details about the values and estimation methods for each parameter can be found in S1 Appendix. Detailed model information, and S2 Appendix. Model parameters.

The diagnostic accuracy for each surveillance method was distinguished between early-stage and late-stage HCC. This was important because early-stage HCC was associated with more effective treatment options, which improved survival. Compliance to surveillance was estimated to be 52% based upon reported rates in clinical practice [43]. Several scenarios with different compliance rates were explored. Diagnostic performance data for ‘US + AFP’ and US (alone) were obtained from the meta-analysis by Tzartzeva et al. (2018) [13]. Different data for the diagnostic accuracy for ‘PIVKA-II + AFP’ was used for cirrhotic and non-cirrhotic patients: non-cirrhotic performance data were obtained from the retrospective study by Pham et al. (2025) [31], and performance data for cirrhotic patients were obtained from Pinjaroen et al. (2026) [30]. Alternative accuracy data for’PIVKA-II + AFP’ from Nan et al. (2024) was also used for scenario analysis [44].

To capture the total costs associated with HCC surveillance, separate costs for detection by early and late-stage HCC should be used. In the absence of stage-specific costs for HCC treatment, this study instead used the average cost per type of first-line HCC treatment, where the treatment received depended upon the HCC stage at diagnosis in the model. A local study reported the distribution of six first-line interventions for early and late HCC detections: OLT, resection, RFA, TACE, supportive care, and systemic treatment [32]. Each intervention was associated with a set survival probability. The distribution of subsequent treatments was estimated based upon data from a local study and a Korean Study, and adjusted to reflect the local first line of treatment [45,46]. For resection, RFA, and TACE, costs were based upon the median cost from the Hospital Authority private charges. Targeted literature searches were used to identify the cost of OLT and supportive care. For systemic treatment, the cost for sorafenib treatment was used.

Patient quality of life (QoL) over the course of the natural disease was reflected in this model by utility values. Utility values were obtained from Zhang et al. [33]. These were derived from 1,071 Chinese patients with chronic Hepatitis B, CLC, DCLC, and different stages of HCC. Since no treatment-specific utility values were reported, the same utility was assumed irrespective of HCC treatment. Each health state of Fig 1 had an associated utility value; a simplifying assumption was that these would not change over time.

### Sensitivity analyses

Several types of sensitivity and scenario analyses were performed to investigate the robustness of the results. This included both one-way sensitivity analysis for identifying key model drivers and probabilistic sensitivity analysis (PSA) for assessing the overall uncertainty of results. Further, targeted scenario analyses were performed for key model settings and parameters, as well as structural assumptions of the model. Detailed results from sensitivity and scenario analyses are presented in S4 Appendix. Sensitivity analyses.

## Results

### Base case results

Simulations estimated that over the course of routine HCC surveillance, a total of 1,249 HCC cases developed per 10,000 individuals. ‘PIVKA-II + AFP’ detected 676 of these (54.1%) during early-stage HCC (BCLC stages 0 or A) [4], compared to 619 (49.5%) for ‘US + AFP’; an increase by 57 cases (4.6 percentage points) per 10,000 surveilled individuals. While this detection rate may appear modest, it reflects the already low surveillance compliance in practice. Compared to ‘US + AFP’, ‘PIVKA-II + AFP’ was associated with a 7.2% decrease in false positive diagnoses. The diagnostic outcomes for each surveillance method are presented in Table 2.

**Table 2:** Diagnostic, cost, health and cost-effectiveness outcomes for bi-annual HCC surveillance methods in a mixed population of LC and CHB patients.

	US + AFP	PIVKA-II + AFP	US (alone)
**Diagnostic outcomes (per 10,000 individuals enrolled in HCC surveillance)**			
Total cases	1,249	1,249	1,249
Detected early stage	619	676	518
Detected late stage	110	90	125
Detected incidentally	39	38	50
Detected symptomatically	481	444	556
% of early detection	49.5%	54.1%	41.5%
True positive	729	766	643
True negative	107,459	108,934	117,707
False negative	243	160	414
False positive	20,513	19,038	10,264
**Total cost and health outcomes (per individual)**			
Total costs (HK $)	211,318	207,174	199,061
Total life years	11.96	11.99	11.91
Total QALYs	9.030	9.049	8.998
**Cost-effectiveness (per individual)**			
	**Incremental costs (HK $)**	**Incremental QALYs**	**ICER per QALY gained (HK $)**
‘PIVKA-II + AFP’ vs. ‘US + AFP’	−4,144	0.019	Dominant
‘PIVKA-II + AFP’ vs. US (alone)	8,112	0.051	157,761

Abbreviations: AFP: alpha-fetoprotein; CHB: chronic hepatitis B; LC: Liver cirrhosis; HCC: hepatocellular carcinoma; ICER: incremental cost-effectiveness ratio; PIVKA-II: protein induced by vitamin K absence or antagonist-II; US: ultrasound.

The average lifetime cost, average QALYs, and cost-effectiveness results for the base-case are shown in Table 2. The overall cost associated with HCC surveillance, management, and treatment per individual enrolled in routine HCC surveillance was estimated to HK $ 207,174 for ‘PIVKA-II + AFP’, compared to HK $ 211,318 for ‘US + AFP; a cost-saving of HK $ 4,144. A breakdown of costs is presented in S3 Appendix. Extended results. The main reasons for the cost-savings compared to the current recommended standard-of-care were the lower cost per surveillance session in the absence of ultrasound assessment, and the reduction in false positive diagnoses and the associated (unnecessary) confirmatory testing.

For health outcomes, ‘PIVKA-II + AFP’ was associated with 11.99 life years, and 9.049 QALYs per individual, compared to 11.96 life years and 9.030 QALYs for ‘US + AFP’; an increase by 0.030 life years and 0.019 QALYs. In terms of cost-effectiveness, ‘PIVKA-II + AFP’ was a dominant strategy under our model scenarios compared to ‘US + AFP’, estimated to both improve health outcomes and decrease overall costs to the Hong Kong healthcare system.

When compared to ultrasound as a standalone tests, ‘PIVKA-II + AFP’ was estimated to increase overall costs per surveilled individual by HK $ 8,112. The QALYs gained from ‘PIVKA-II + AFP’ vs. US (alone) was estimated to 0.051. This suggests that ‘PIVKA-II + AFP’ would be cost-effective compared to US (alone), with an ICER per QALY gained of HK $ 157,761.

### Sub-group analysis

To further understand which patients could benefit most from biomarker-based HCC surveillance using ‘PIVKA-II + AFP’, a sub-group analysis was also performed for cirrhotic vs. non-cirrhotic patients. Two separate model simulations were performed for one target population, including exclusively non-cirrhotic CHB patients, and one exclusively for LC patients. Full results are shown in S3 Appendix. Extended results. This analysis showed that the incremental benefit of ‘PIVKA-II + AFP’ compared to ‘US + AFP’ is greatest when targeting non-cirrhotic patients. In this group, the prevalence of HCC was lower, and QALYs were highly similar as a result, only slightly favoring ‘PIVKA-II + AFP’. However, compared to ‘US + AFP’, ‘PIVKA-II + AFP’ reduced the number of false positive diagnoses by 17%, due to its suggested higher specificity, thereby alleviating many costs associated with confirmatory diagnoses. By contrast, in cirrhotic patients, ‘PIVKA-II + AFP’ offered a larger improvement in the early-stage HCC detection rate, but also raised overall costs due to an increased number of false positive diagnoses, due to the suggested slightly lower diagnostic specificity of PIVKA and AFP in this cohort. Compared to ‘US + AFP’, ‘PIVKA-II + AFP’ was cost-effective for cirrhotic patients, with an ICER per QALY gained of HK $ 112,386. The difference in the relative value of ‘PIVKA-II + AFP’ between cirrhotic and non-cirrhotic patients is largely explained by the suggested difference in the diagnostic performance for the two populations. This is important to note since uncertainties in diagnostic performance due to the sourcing of results from different populations represent a major limitation of this evaluation (as will be further discussed in the limitation section). However, in this context, it is to emphasise that it has not been the objective of this analysis to identify dominance and superiority but rather to discuss the conditions under which PIVKA and AFP may be considered non-inferior compared to AFP and US using modeling techniques. To address data uncertainties, scenario and sensitivity analysis were employed to further validate the robustness of the conclusions from these evaluations.

### Scenario and sensitivity analyses

The sensitivity and scenario analyses showed that results from the base case were relatively robust. As described above, the choice of target population for HCC surveillance was important for the results, with ‘PIVKA-II + AFP’ showing more favorable cost-effectiveness outcomes when targeting non-cirrhotic patients; within this patient group ‘PIVKA-II + AFP’ was also associated with lower false positive diagnoses and the associated costs for unnecessary confirmatory testing. However, all scenarios still resulted in a relatively good cost-effectiveness ratio far below the 1 time GDP and with marginal variance in the relevant model outputs. The cost of ultrasound and PIVKA-II were impactful for results, as well as whether screening using ‘PIVKA-II + AFP’ could improve patient adherence to surveillance. ‘PIVKA-II + AFP’ remained dominant in the scenario analysis based upon the more pessimistic diagnostic performance data for from Nan et al. as well [44]. Overall, ‘PIVKA-II + AFP’ remained dominant or highly cost-effective across all scenarios, suggesting that the findings from this study were relatively robust to changes in the data used for the analysis.

The one-way sensitivity analysis and scenario analyses identified the most impactful parameters for the model’s results as: HCC and DCLC incidence rates, the cost of the HCC screening tests, the cost of false positive diagnoses (incl. the cost of confirmatory testing), and patient compliance to surveillance. The probabilistic sensitivity analysis (PSA) confirmed that the combination of PIVKA-II plus AFP was the preferred method for HCC surveillance from a cost-effectiveness perspective, even when considering parametric uncertainty.

## Discussion

This study has used economic modelling to explore the value of ‘PIVKA-II + AFP’ for HCC surveillance in patients with compensated liver cirrhosis or chronic hepatitis B infection in Hong Kong. The results suggest that HCC surveillance utilizing ‘PIVKA-II + AFP’ could be a valuable alternative for patients lacking access to ultrasound. Compared to ‘US + AFP’ – the recommended standard-of-care in Hong Kong – surveillance using ‘PIVKA-II + AFP’ was estimated to increase the share of HCC cases detected during early-stage from 49.5% to 54.1%. Biomarker-based surveillance could reduce the dependence on ultrasonography assessment, currently a bottleneck of HCC assessment in Hong Kong due to long waiting times [47].

For a mixed population of patients with non-cirrhotic CHB and those with LC, ‘PIVKA-II + AFP’ was suggested to be associated with lower overall costs for the Hong Kong healthcare system than ‘US + AFP’, with a projected cost saving of HK $ 4,144 per patient. In terms of patient health, ‘PIVKA-II + AFP’ was associated with better outcomes than ‘US + AFP’, with an estimated 0.019 QALYs gained per patient. From a cost-effectiveness perspective, this means that ‘PIVKA-II + AFP’ is estimated to be dominant compared to the current recommended standard-of-care, i.e., associated with better health outcomes for patients whilst also being cost-saving. Earlier cost-effectiveness studies in Hong Kong and mainland China have also reported similar conclusions, demonstrating that ‘PIVKA‑II + AFP’ is cost‑effective compared with ultrasound alone, with an ICER of USD 5,185.89 per QALY [44,48]. The results suggested that the relative value of ‘PIVKA-II + AFP’ is greatest when targeting non-cirrhotic patients. When targeting cirrhotic patients, ‘PIVKA-II + AFP’ was still estimated to be cost-effective compared to ‘US + AFP’, though it was associated with higher overall costs due to a substantial increase in false positive diagnoses.

The model’s results were most sensitive towards changes to HCC incidence, DCLC incidence, the cost of false positive diagnoses, starting age of surveillance, and the cost of surveillance using ‘US + AFP’. ‘PIVKA-II + AFP’ was cost-effective across all age ranges, but its relative value was highest for patients aged 40–60 years. When compared to US assessment as a standalone test, ‘PIVKA-II + AFP’ was estimated to be highly cost-effective among both cirrhotic and non-cirrhotic patients. The results of this study should be interpreted with caution, given the nature of the modelling methodology and assumptions and carefully considered in the context of the prevailing body of evidence and clinical opinion.

It is important to acknowledge that additional factors and variables may inform decision-making in clinical practice, and this study does not suggest that ‘PIVKA-II + AFP’ should replace ultrasound-based assessment as the standard-of-care. However, it shows that biomarker-based assessment using ‘PIVKA-II + AFP’ could serve as a viable alternative for Hong Kong patients with limited access to ultrasound. Since results from biomarker-based assessment are not dependent upon the skills of the test performer, such surveillance strategies may support the scalability of surveillance programs. Furthermore, PIVKA-II-based HCC surveillance may have the potential to reduce the number of false positive diagnoses among non-cirrhotic patients, which could alleviate diagnostic resources used for confirmatory testing. The prospects of adopting ‘PIVKA-II + AFP’ into Hong Kong routine HCC surveillance are relatively good. Many Hong Kong hospitals already have access to the necessary infrastructure to adopt the technology, including the laboratory capacity and familiarity among clinicians. AFP is already routinely utilised within HCC surveillance as part of the recommended standard of care (‘US + AFP’).

There is potential for better diagnostic performance through the combination of PIVKA-II and AFP with ultrasound or other markers. While data on the combined performance of ultrasound and ‘PIVKA-II + AFP’ is not yet available, studies suggest that the clinically validated digital algorithm such as GAAD – which combines PIVKA-II, AFP, and the patient characteristics of age and gender – may improve diagnostic performance [49,50]. Cost-effectiveness studies from mainland China and Thailand suggested that suggested that GAAD – both by itself and combined with ultrasound – have the potential to further improve cost-effectiveness as well [30,44].

To capture the value of HCC surveillance, it is crucial to distinguish the diagnostic performance between early-stage HCC and that of all-stage HCC. Diagnostic accuracy data for US (alone) and US + AFP were obtained from the meta-analysis by Tzartzeva et al. [13], the only identified meta-analysis that reported separate accuracy data for early-stage and all-stage HCC. A limitation of this study is the absence of head-to-head comparison between ‘PIVKA-II + AFP’ and the US-based methods. This means that differences in diagnostic performance between surveillance methods may be influenced by between-study heterogeneity, including differences in study design and patient populations. To assess the potential impact from this, scenario analyses with more conservative diagnostic accuracy data from Nan et al. [44] was used. These confirmed that the findings from the study about HCC surveillance with ‘PIVKA-II + AFP’ yielding non-inferior results to the recommended standard-of-care are relatively robust [51].

In the absence of method-specific compliance data, this analysis assumed identical compliance rates to HCC surveillance regardless of assessment method. In clinical practice, however, surveillance methods that do not require ultrasonography may be easier to offer at a suitable time for the patient and hence are likely to see higher compliance. For this reason, it is possible that this study has slightly underestimated the relative benefits of PIVKA-II to the Hong Kong medical system.

Future studies should consider collecting head-to-head comparison data between PIVKA-II-based and US-based HCC surveillance methods, particularly in early-stage HCC detection, to provide more robust evidence. It should also evaluate the performance of combining PIVKA-II and AFP with ultrasound, as well as whether other biomarkers or combinations of biomarkers, such as GAAD and GALAD, could provide even better diagnostic accuracy and cost-effectiveness.

In the absence of data on wider societal costs associated with HCC screening and treatment, this study adopted a healthcare payer perspective. It is likely that the benefit from a biomarker-based approach would be greater if patients’ time and costs for screening and confirmatory testing could have been accounted for. Some of the findings from this study – e.g., the potential of PIVKA-II-based screening to reduce false positive diagnoses among non-cirrhotic patients – could likely be generalized to other similar healthcare systems with limited ultrasound capacity, though it is important to first evaluate cost-effectiveness within each healthcare system.

In conclusion, the adoption of biomarker-based surveillance into routine HCC surveillance has the potential to improve the detection of early-stage HCC in Hong Kong, enabling access to effective treatment options and survival gains for patients. Compared to the current recommended standard-of-care in Hong Kong, screening using ‘PIVKA-II + AFP’ could reduce false positive diagnoses, confirmatory testing, and unwarranted patient concerns. It also offers a valuable option to ultrasound-based HCC surveillance where healthcare capacity is constrained.

## Supporting information

S1 Appendix Detailed model information. (DOCX)

S2 Appendix Model parameters. (DOCX)

S3 Appendix Extended results. (DOCX)

S4 Appendix Sensitivity analyses. (DOCX)
