# Supplementary material for: Surveillance of hepatocellular carcinoma (HCC) patients using Protein Induced by Vitamin K (PIVKA-II): A cost-utility analysis for Hong Kong
Source: PLoS One. 2026 Jul 17;21(7):e0353882. doi: 10.1371/journal.pone.0353882 (PMC13378965; doi:10.1371/journal.pone.0353882)
Supplement: S1 Appendix — (DOCX) [file pone.0353882.s001.docx]

# S1 Appendix: Detailed model information

**Model structure**

For each 6-month cycle, screened individuals can remain in the current state or transition to another, subject to conditional probabilities. Screened individuals either enter the model from CHB or LC, at a pre-defined mix of 85% CHB and 15% LC. Those with CHB can either remain in “CHB”, developed to “LC”, “HCC 0/A” or “Death”. Similarly, individuals with LC, could either remain in “LC” or progress to “DCLC” (decompensated liver cirrhosis), “HCC 0/A” or “Death”.

The positive tested patients from HCC surveillance undergo a confirmatory test by means of computed tomography (CT). This includes both patients with HCC (true positive diagnoses) and without HCC (false positive). For simplicity, it was assumed that the confirmatory test is 100% accurate. For HCC-positive individuals not detected through routine surveillance (i.e. false negative diagnoses), incidental or symptomatic detection of HCC is possible, to reflect a real-world setting where patients may seek medical care even where routine HCC surveillance has not indicated a problem. The chance of symptomatic detection is higher for late-stage HCC (Stage B/C/D) than in early-stage HCC (Stage 0/A). If individuals develop early HCC and the tumor is detected in the initial state, interventions take place and individuals can receive one of six possible treatments [1]:

- Orthotopic liver transplantation (OLT). Before patients received a liver transplant, it is assumed that they stayed one cycle (6 months) on the waiting list for transplantation.
- ‘Resection’ defined by hepatic resection to remove the tumor and the time after resection.
- Radiofrequency thermal ablation (RFA)
- Transarterial Chemoembolization (TACE)
- Systemic therapy (Sorafenib)
- Best supportive care

Once entered into treatment, patients stay in this health-state until death; their survival is then conditional upon the survival curve for the specific treatment. Due to data limitations, once entered into treatment, no distinction is made by the preceding health condition. This means that whilst the HCC stage at detection – early-stage (0/A) vs. late-stage (B/C/D) – affects the probability of receiving a certain type of treatment, the treatment-specific survival itself is the same for all patients receiving that treatment.

Patients who develop decompensated liver cirrhosis (DCLC), can remain in this health state until they either receive OLT or die. Individuals in the ‘DCLC’ state are assumed to automatically be placed on the waitlist for OLT. If individuals in the initial state develop early HCC (BCLC 0/A) but are not detected in surveillance, they progress to the state ‘HCC 0/A undetected’. Individuals in ‘HCC 0/A Undetected’ can also transition into ‘HCC B/C/D Undetected’, where the tumor is defined by the BCLC stages B, C, or D. Another possibility is to develop decompensated cirrhosis and progress to the ‘DCLC’ state. Another possibility for HCC being detected, apart from surveillance, is the incidental diagnosis when patients present with symptoms. This incidental diagnosis can happen in the previously mentioned four states and entails the same transition options as diagnosis in surveillance. As long as the tumor remains undetected, and individuals do not progress to ‘Death’ due to the disease or other causes they can remain in ‘HCC B/C/D undetected’. If HCC is detected in surveillance, a late detection intervention can take place and individuals progress to the state of ’Advanced stage treatment’ where they can remain until they die due to the disease or other causes. ‘Death’ is an absorbing state which means that individuals remain in it once they have entered.

**Cost calculations**

The cost of delivering laboratory diagnostics consists of three parts: the price of the biomarker test or tests to the Hospital Authority (which is confidential per local practice), the salary of the nurse (who draws the blood) and the salary of laboratory technician (who runs the test or tests). When estimating a nurse’s salary, we referenced the highest hourly salary of a registered nurse from Hospital Authority. The number of patients a nurse can serve in an hour was based on a local study, which shows that it takes 10.4 minutes to complete a job.(Leung et al. 2006) We estimated that a technician can do 150 tests in two hours based on our understanding of the workflow of a typical medical laboratory. We used this cost structure for ‘PIVKA II + AFP’, with the delivery cost estimated at HK$ 224.06.

The cost of systemic treatment is most challenging due to the evolving treatment landscape and confidential drug prices. The commonly used treatment is the tyrosine kinase inhibitor Sorafenib, which is in the Community Care Fund Medical Medical Assistance Programme for treating liver cancer. From their report, we can obtain the subsidy per applicant for Sorafenib [2].

# References

1. Chui AMN, Yau TCC, Cheung TT (2020) An overview in management of hepatocellular carcinoma in Hong Kong using the Hong Kong Liver Cancer (HKLC) staging system. Glob Health Med 2:312–318

2. Hospital Authority (2025) CCF Medical Assistance Programmes Cumulative number of approved applications & subsidy amount.
