# Supplementary material for: Surveillance of hepatocellular carcinoma (HCC) patients using Protein Induced by Vitamin K (PIVKA-II): A cost-utility analysis for Hong Kong
Source: PLoS One. 2026 Jul 17;21(7):e0353882. doi: 10.1371/journal.pone.0353882 (PMC13378965; doi:10.1371/journal.pone.0353882)
Supplement: S2 Appendix — (DOCX) [file pone.0353882.s002.docx]

# S2 Appendix: Model parameters

All input data for the health economic model is shown in Table 1. Where 95% confidence intervals were presented in literature, or could readily be calculated from the data provided, these values were used as the lower and upper limits for the probabilistic and one-way sensitivity analyses. Where no data was available beyond the mean/point estimate, lower and upper limits were calculated as the base case estimate ±20%.

Costs of direct healthcare services, including surveillance, confirmatory diagnostics, treatments, and prognosis, were extracted from local publications. All costs are reported in HK$ (HK$1 = US$0.13). These were inflation-adjusted inflated to the value of year 2024 using CPI inflation rate [1].

Table 1: Overview of model inputs for the economic analysis, base case

| **Parameter** | **Base  value** | **Lower plausible range** | **Upper plausible range** | **Source** |
| --- | --- | --- | --- | --- |
| **Age of surveillance** | | | | |
| Age at start of surv, LC (ALD) | 56 | 46 | 66 | [Fung, et al. 2007](https://journals.lww.com/eurojgh/abstract/2007/08000/etiologies_of_chronic_liver_diseases_in_hong_kong.8.aspx) [2] |
| Age at start of surv, LC (HBV) | 45 | 34 | 53 |  |
| Age at start of surv, LC (HCV) | 56 | 46 | 66 |  |
| Age at start of surv, LC (NAFLD) | 53 | 44 | 62 |  |
| Age at start of surv, NC.CHB | 45 | 34 | 53 |  |
| Age upper limit for the surv. | 70* | 65 | 75 | Assumption |
| **Patient type distribution** | | | | |
| Percentage in surveillance with LC | 15.0% | 15.0% | 15.0% | Assumption |
| Percentage in surveillance with NC.CHB | 85.0% | 85.0% | 85.0% | Assumption |
| **Aetiology for LC patients** | | | | |
| Aetiology (ALD) | 1.8% | 1.8% | 1.8% | [Fung, et al. 2007](https://journals.lww.com/eurojgh/abstract/2007/08000/etiologies_of_chronic_liver_diseases_in_hong_kong.8.aspx) [2] |
| etiology (HBV) | 91.5% | 91.5% | 91.5% |  |
| Aetiology (HCV) | 5.2% | 5.2% | 5.2% |  |
| Aetiology (NAFLD) | 1.6% | 1.6% | 1.6% |  |
| **Incidence rates** | | | | |
| HCC, incidence rate annual if CHB | 0.31% | 0.25% | 0.37% | [Wong, et al. 2018](https://www.nature.com/articles/s41575-018-0055-0) [3] |
| HCC, incidence rate annual if ALD | 0.47% | 0.38% | 0.56% | [Wong, et al. 2018](https://www.nature.com/articles/s41575-018-0055-0) [3] |
| HCC, incidence rate annual if HBV | 2.19% | 1.75% | 2.63% | [Wong, et al. 2018](https://www.nature.com/articles/s41575-018-0055-0) [3] |
| HCC, incidence rate annual if HCV | 0.35% | 0.28% | 0.42% |  |
| HCC, incidence rate annual if NAFLD | 1.09% | 0.87% | 1.31% |  |
| LC, incidence rate annual if CHB | 0.83% | 0.66% | 1.00% |  |
| DCLC, incidence rate annual if ALD | 7.3% | 6.5% | 8.2% | [NICE guideline NG50](https://www.nice.org.uk/guidance/ng50/evidence/appendices-iq-pdf-2546540173), [4] [Fleming 2010](https://pubmed.ncbi.nlm.nih.gov/21050236/) [5] |
| DCLC, incidence rate annual if HBV | 5.0% | 4.0% | 6.0% | [NICE guideline NG50](https://www.nice.org.uk/guidance/ng50/evidence/appendices-iq-pdf-2546540173), [4] [Dakin 2010](https://pubmed.ncbi.nlm.nih.gov/20825619/) [6] |
| DCLC, incidence rate annual if HCV | 4.0% | 3.2% | 4.8% | [NICE guideline NG50](https://www.nice.org.uk/guidance/ng50/evidence/appendices-iq-pdf-2546540173), [4] [Wright 2006](https://researchonline.lshtm.ac.uk/id/eprint/11636/1/FullReport-hta10210.pdf) [7] |
| DCLC, incidence rate annual if NAFLD | 3.8% | 3.0% | 4.5% | [Chris Estes 2017](https://doi.org/10.1002/hep.29466) [8] |
| **Incidental detection rate** | | | | |
| Annual incidental detection HCC (BCLC stage: 0/A) | 6.9% | 0.0% | 23.3% | [Thompson-Coon et al, 2007](https://pubmed.ncbi.nlm.nih.gov/17767898/) [9] |
| Annual incidental detection HCC (BCLC stage: B/C/D) | 31.1% | 0.0% | 65.2% | [Thompson-Coon et al, 2007](https://pubmed.ncbi.nlm.nih.gov/17767898/) [9] |
| Patients with DCLC after HCC treatment | 15.5% | 12.4% | 18.6% | [Kondo et al, 2022](https://journals.plos.org/plosone/article?id=10.1371/journal.pone.0261619) [10] |
| **HCC treatment distribution for detection during early-stage HCC (BCLC stages 0 & A)** | | | | |
| OLT_early | 3.5% | 3.5% | 3.5% | [Yau et al. 2014](https://linkinghub.elsevier.com/retrieve/pii/S0016508514002431) [11] |
| Resection_early | 45.6% | 45.6% | 45.6% | [Yau et al. 2014](https://linkinghub.elsevier.com/retrieve/pii/S0016508514002431) [11] |
| RFA_early | 29.7% | 29.7% | 29.7% | [Yau et al. 2014](https://linkinghub.elsevier.com/retrieve/pii/S0016508514002431) [11] |
| TACE_early | 16.9% | 16.9% | 16.9% | [Yau et al. 2014](https://linkinghub.elsevier.com/retrieve/pii/S0016508514002431) [11] |
| Systemic Treatment_early | 1.51% | 1.51% | 1.51% | [Yau et al. 2014](https://linkinghub.elsevier.com/retrieve/pii/S0016508514002431) [11] |
| BSC_early | 2.8% | 2.8% | 2.8% | [Yau et al. 2014](https://linkinghub.elsevier.com/retrieve/pii/S0016508514002431) [11] |
| **HCC treatment distribution for detection during late-stage HCC (BCLC stages B, C & D)** | | | | |
| OLT_late | 2.7% | 2.7% | 2.7% | [Yau et al. 2014](https://linkinghub.elsevier.com/retrieve/pii/S0016508514002431) [11] |
| Resection_late | 21.1% | 21.1% | 21.1% |  |
| RFA_late | 2.5% | 2.5% | 2.5% |  |
| TACE_late | 28.0% | 28.0% | 28.0% |  |
| Systemic Treatment_late | 21.4% | 21.4% | 21.4% |  |
| BSC_late | 24.3% | 24.3% | 24.3% |  |
| **Waiting list for OLT** | | | | |
| OLT waiting time (in 6-mo cycles) | 7 | - | - | [Legislative Council Secretariat, 2021](https://www.legco.gov.hk/research-publications/english/2021issh15-organ-donation-20210114-e.pdf) [12] |
| DCLC, listed for OLT | 5% | 4% | 6% | Assumption |
| **Adherence to surveillance** | | | | |
| Compliance rate, US+AFP | 52% | 38% | 63% | [Zhao et al, 2018](https://pubmed.ncbi.nlm.nih.gov/28834146/) [13] |
| Compliance rate, PIVKA II+AFP | 52% | 38% | 63% |  |
| Compliance rate, US | 52% | 38% | 63% |  |
| **Utilities (quality of life values)** | | | | |
| (QoL) LC | 0.750 | 0.724 | 0.776 | [Zhang et al. 2021](https://pubmed.ncbi.nlm.nih.gov/34526336/) [14] |
| (QoL) Non-Cirrhotic Chronic Hepatitis B | 0.773 | 0.763 | 0.783 | [Zhang et al. 2021](https://pubmed.ncbi.nlm.nih.gov/34526336/) [14] |
| (QoL) DCLC | 0.683 | 0.656 | 0.710 | [Zhang et al. 2021](https://pubmed.ncbi.nlm.nih.gov/34526336/) [14] |
| (QoL) HCC undetected | 0.640 | 0.623 | 0.657 | [Zhang et al. 2021](https://pubmed.ncbi.nlm.nih.gov/34526336/) [14] |
| (QoL) WL | 0.640 | 0.623 | 0.657 | [Zhang et al. 2021](https://pubmed.ncbi.nlm.nih.gov/34526336/) [14] |
| (QoL) OLT & Post | 0.640 | 0.623 | 0.657 | [Zhang et al. 2021](https://pubmed.ncbi.nlm.nih.gov/34526336/) [14] |
| (QoL) Resection and Post | 0.640 | 0.623 | 0.657 | [Zhang et al. 2021](https://pubmed.ncbi.nlm.nih.gov/34526336/) [14] |
| (QoL) RFA & Post | 0.640 | 0.623 | 0.657 | [Zhang et al. 2021](https://pubmed.ncbi.nlm.nih.gov/34526336/) [14] |
| (QoL) TACE & Post | 0.640 | 0.623 | 0.657 | [Zhang et al. 2021](https://pubmed.ncbi.nlm.nih.gov/34526336/) [14] |
| (QoL) BSC & Post | 0.615 | 0.581 | 0.649 | [Zhang et al. 2021](https://pubmed.ncbi.nlm.nih.gov/34526336/) [14] |
| (QoL) Systemic treatment | 0.640 | 0.623 | 0.657 | [Zhang et al. 2021](https://pubmed.ncbi.nlm.nih.gov/34526336/) [14] |
| (QoL) Palliative | 0.615 | 0.581 | 0.649 | [Zhang et al. 2021](https://pubmed.ncbi.nlm.nih.gov/34526336/) [14] |
| **Surveillance costs** | | | | |
| US+AFP | HK $ 691 | HK $ 553 | HK $ 829 | [Yuen et al. 2000](https://pubmed.ncbi.nlm.nih.gov/10655254/) [15], adjusted for inflation |
| Elecsys PIVKA II + Elecsys AFP | HK $ 224.06 | HK $ 179 | HK $ 269 | Roche diagnostics, data on file |
| US | HK $ 553 | HK $ 442 | HK $ 663 | [Yuen et al. 2000](https://pubmed.ncbi.nlm.nih.gov/10655254/) [15] , adjusted for inflation |
| **Event costs** | | | | |
| LC, annual | HK $ 20,500 | HK $ 16,400 | HK $ 24,600 | Leung et al. (2023), inflation adjusted and, verified by KOL [16] |
| NC.CHB, annual (other than surveillance) | HK $ 0 | HK $ 0 | HK $ 0 | Leung et al. (2023), inflation adjusted and, verified by KOL [16] |
| DCLC, annual | HK $ 0 | HK $ 0 | HK $ 0 | Leung et al. (2023), inflation adjusted and, verified by KOL [16] |
| True positive for HCC (confirmatory) | HK $ 116,248 | HK $ 92,998 | HK $ 139,498 | Leung et al. (2023), inflation adjusted and, verified by KOL [16] |
| False positive for HCC | HK $ 14,018 | HK $ 11,214 | HK $ 16,821 | Leung et al. (2023), inflation adjusted and, verified by KOL [16] |
| Incidental diagnosis | HK $ 10,228 | HK $ 8,182 | HK $ 12,273 | Leung et al. (2023), inflation adjusted and, verified by KOL [16] |
| Follow-up after HCC, per cycle | HK $ 5,296 | HK $ 4,236 | HK $ 6,355 | Leung et al. (2023), inflation adjusted and, verified by KOL [16] |
| **Treatment costs** | | | | |
| OLT, per operation | HK $ 1,206,152 | HK $ 964,921 | HK $ 1,447,382 | Local costing data on file, verified by KOL [16] |
| Post-OLT follow up (year 1) | HK $ 712,601 | HK $ 570,081 | HK $ 855,121 |  |
| Post-OLT follow up (year 2+) | HK $ 112,896 | HK $ 90,317 | HK $ 135,475 |  |
| Resection | HK $ 121,760 | HK $ 97,408 | HK $ 146,112 |  |
| RFA | HK $ 56,430 | HK $ 45,144 | HK $ 67,716 |  |
| TACE | HK $ 56,487 | HK $ 45,189 | HK $ 67,784 |  |
| BSC, per month | HK $ 17,586 | HK $ 14,069 | HK $ 21,103 |  |
| Systemic treatment, annual | HK $ 51,574 | HK $ 41,259 | HK $ 61,888 |  |
| **Analytical settings** | | | | |
| Discount rate | 3.0% | - | - | [Home Affairs Bureau, 2008](https://www.legco.gov.hk/yr07-08/english/fc/pwsc/papers/p08-31e.pdf) [17] |
| Willingness to pay (CE Threshold) | HK$422,242 | HK$422,242 | HK$422,242 | [Census and Statistics Department, 2024](https://www.censtatd.gov.hk/en/web_table.html?id=310-31001) [18] |
| **Diagnostic accuracy** | | | | |
| US (for all population) | | | | |
| Sensitivity, US, early | 45.0% | 30.0% | 62.0% | [Tzartzeva K, et al. 2018](https://pubmed.ncbi.nlm.nih.gov/29425931/) [19] |
| Specificity, US, early | 92.0% | 85.0% | 96.0% | [Tzartzeva K, et al. 2018](https://pubmed.ncbi.nlm.nih.gov/29425931/) [19] |
| Sensitivity, US, all | 78.0% | 67.0% | 86.0% | [Tzartzeva K, et al. 2018](https://pubmed.ncbi.nlm.nih.gov/29425931/) [19] |
| Specificity, US, all | 92.0% | 85.0% | 96.0% | [Tzartzeva K, et al. 2018](https://pubmed.ncbi.nlm.nih.gov/29425931/) [19] |
| US+AFP (for all population) | | | | |
| Sensitivity, US+AFP, early | 63.0% | 48.0% | 75.0% | [Tzartzeva K, et al. 2018](https://pubmed.ncbi.nlm.nih.gov/29425931/) [19] |
| Specificity, US+AFP, early | 84.0% | 77.0% | 89.0% | [Tzartzeva K, et al. 2018](https://pubmed.ncbi.nlm.nih.gov/29425931/) [19] |
| Sensitivity, US+AFP, all | 97.0% | 91.0% | 99.0% | [Tzartzeva K, et al. 2018](https://pubmed.ncbi.nlm.nih.gov/29425931/) [19] |
| Specificity, US+AFP, all | 84.0% | 77.0% | 89.0% | [Tzartzeva K, et al. 2018](https://pubmed.ncbi.nlm.nih.gov/29425931/) [19] |
| PIVKA-II+AFP (LC population) | | | | |
| Sensitivity, PIVKA II+AFP, early | 75.6% | 67.4% | 82.5% | [Chan et al, 2022](https://medically.gene.com/global/en/unrestricted/oncology/diseases/gastrointestinal-cancer/materials/elecsys-pivka-ii-and-elecsys-afp-assays-demonstrate-goo.html) [20], [Pinjaroen et al. 2026](https://journals.plos.org/plosone/article?id=10.1371/journal.pone.0337913) [21] |
| Specificity, PIVKA II+AFP, early | 75.9% | 66.9% | 83.5% | [Chan et al, 2022](https://medically.gene.com/global/en/unrestricted/oncology/diseases/gastrointestinal-cancer/materials/elecsys-pivka-ii-and-elecsys-afp-assays-demonstrate-goo.html), [20] [Pinjaroen et al. 2026](https://journals.plos.org/plosone/article?id=10.1371/journal.pone.0337913) [21] |
| Sensitivity, PIVKA II+AFP, all | 86.8% | 82.3% | 90.5% | [Chan et al, 2022](https://medically.gene.com/global/en/unrestricted/oncology/diseases/gastrointestinal-cancer/materials/elecsys-pivka-ii-and-elecsys-afp-assays-demonstrate-goo.html), [20] [Pinjaroen et al. 2026](https://journals.plos.org/plosone/article?id=10.1371/journal.pone.0337913) [21] |
| Specificity, PIVKA II+AFP, all | 75.9% | 66.9% | 83.5% | [Chan et al, 2022](https://medically.gene.com/global/en/unrestricted/oncology/diseases/gastrointestinal-cancer/materials/elecsys-pivka-ii-and-elecsys-afp-assays-demonstrate-goo.html), [20] [Pinjaroen et al. 2026](https://journals.plos.org/plosone/article?id=10.1371/journal.pone.0337913) [21] |
| PIVKA-II+AFP (CHB population, base case) | | | | |
| Sensitivity, PIVKA II+AFP, early | 82.1% | 74.0% | 90.3% | [Pham et al. (2025)](https://link.springer.com/article/10.1007/s12672-025-03651-4) [22] |
| Specificity, PIVKA II+AFP, early | 96.0% | 95.2% | 96.6% | [Pham et al. (2025)](https://link.springer.com/article/10.1007/s12672-025-03651-4) [22] |
| Sensitivity, PIVKA II+AFP, all | 84.4% | 78.1% | 90.7% | [Pham et al. (2025)](https://link.springer.com/article/10.1007/s12672-025-03651-4) [22] |
| Specificity, PIVKA II+AFP, all | 96.0% | 95.2% | 96.6% | [Pham et al. (2025)](https://link.springer.com/article/10.1007/s12672-025-03651-4) [22] |
| PIVKA-II+AFP (CHB population, alternative data for scenario analysis) | | | | |
| Sensitivity, PIVKA II+AFP, early | 76.1% | 68.5% | 83.7% | [Nan et al. (2024)](https://pubmed.ncbi.nlm.nih.gov/38415341/) [23] |
| Specificity, PIVKA II+AFP, early | 84.0% | 75.6% | 92.4% | [Nan et al. (2024)](https://pubmed.ncbi.nlm.nih.gov/38415341/) [23] |
| Sensitivity, PIVKA II+AFP, all | 90.7% | 81.6% | 99.8% | [Nan et al. (2024)](https://pubmed.ncbi.nlm.nih.gov/38415341/) [23] |
| Specificity, PIVKA II+AFP, all | 84.0% | 75.6% | 92.4% | [Nan et al. (2024)](https://pubmed.ncbi.nlm.nih.gov/38415341/) [23] |

*The upper age limit for HCC surveillance is not specified in Hong Kong clinical guidelines, but clinical experts confirmed that 70 years was a feasible age for the base case. The impact of changing the upper age limit of the target population was assessed through scenario analyses.

Abbreviations: AFP: alpha-fetoprotein; CHB: chronic hepatitis B; LC: Liver cirrhosis; HCC. Hepatocellular carcinoma; PIVKA-II: protein induced by vitamin K absence or antagonist-II; QoL: quality of life; WL: waiting list

Table 2: Estimated survival by model health state and treatments

| Health state / treatment | 1-year survival (%) | 5-year survival (%) | Median survival (months) | Source |
| --- | --- | --- | --- | --- |
| LC | 95.0 | 77.0 | 174 | [D’Amico et al, 2006](https://pubmed.ncbi.nlm.nih.gov/16298014/) [24] |
| DCLC | 73.0 | 21.0 | 30 | [Trebicka et al, 2020](https://pubmed.ncbi.nlm.nih.gov/32673741/) [25] |
| HCC undetected | 54.0 | 5.0 | 14 | [Khalaf et al, 2017](https://pubmed.ncbi.nlm.nih.gov/27521507/) [26] |
| OLT | 97.0 | 79.0 | 214 | [Wong et al, 2018](https://www.nature.com/articles/s41575-018-0055-0) [3] |
| Resection | 95.8 | 73.0 | 87 | [Yim et al, 2016](https://onlinelibrary.wiley.com/doi/abs/10.1111/liv.12960#support-information-section) [27] |
| RFA | 93.0 | 74.0 | 200 | [Yim et al, 2016](https://onlinelibrary.wiley.com/doi/abs/10.1111/liv.12960#support-information-section) [27] |
| TACE | 52.0 | 18.0 | 13 | [Yim et al, 2016](https://onlinelibrary.wiley.com/doi/abs/10.1111/liv.12960#support-information-section) [27] |
| Systemic treatment | 16.3 | 11.4 | 3 | [Yim et al, 2016](https://onlinelibrary.wiley.com/doi/abs/10.1111/liv.12960#support-information-section) [27] |
| BSC | 17.0 | 1.0 | 3 | [Yim et al, 2016](https://onlinelibrary.wiley.com/doi/abs/10.1111/liv.12960#support-information-section) [27] |
| Non-cirrhotic CHB | 99.0 | 85.0 | 239 | [Fattovich, 2003](https://www.journal-of-hepatology.eu/article/S0168-8278(03)00139-9/fulltext) [28] |

Abbreviations: BSC: Best supportive care; LC = Liver cirrhosis; CHB: Chronic hepatitis B; DCLC = Decompensated liver cirrhosis; HCC = Hepatocellular carcinoma; OLT = Orthotopic liver transplantation; RFA = Radiofrequency thermal ablation; TACE = Transarterial chemoembolization

# References

1. World Bank Open Data [Internet]. [cited 2026 Jan 16]. World Bank Open Data. Available from: https://data.worldbank.org

2. Fung KTT, Fung J, Lai CL, Yuen MF. Etiologies of chronic liver diseases in Hong Kong. Eur J Gastroenterol Hepatol. 2007 Aug;19(8):659. doi:10.1097/MEG.0b013e3281ace0b7

3. Wong MCS, Huang JLW, George J, Huang J, Leung C, Eslam M, et al. The changing epidemiology of liver diseases in the Asia–Pacific region. Nat Rev Gastroenterol Hepatol. 2019 Jan;16(1):57–73. doi:10.1038/s41575-018-0055-0

4. Overview | Cirrhosis in over 16s: assessment and management | Guidance | NICE [Internet]. NICE; 2016 [cited 2025 Jun 18]. Available from: https://www.nice.org.uk/guidance/ng50

5. Fleming KM, Aithal GP, Card TR, West J. The rate of decompensation and clinical progression of disease in people with cirrhosis: a cohort study. Aliment Pharmacol Ther. 2010 Dec;32(11–12):1343–50. doi:10.1111/j.1365-2036.2010.04473.x PubMed PMID: 21050236.

6. Dakin H, Bentley A, Dusheiko G. Cost-utility analysis of tenofovir disoproxil fumarate in the treatment of chronic hepatitis B. Value Health J Int Soc Pharmacoeconomics Outcomes Res. 2010 Dec;13(8):922–33. doi:10.1111/j.1524-4733.2010.00782.x PubMed PMID: 20825619.

7. Wright M, Grieve R, Roberts J, Main J, Thomas HC, UK Mild Hepatitis C Trial Investigators. Health benefits of antiviral therapy for mild chronic hepatitis C: randomised controlled trial and economic evaluation. Health Technol Assess Winch Engl. 2006 Jul;10(21):1–113, iii. doi:10.3310/hta10210 PubMed PMID: 16750059.

8. Estes C, Razavi H, Loomba R, Younossi Z, Sanyal AJ. Modeling the epidemic of nonalcoholic fatty liver disease demonstrates an exponential increase in burden of disease. Hepatology. 2018 Jan;67(1):123. doi:10.1002/hep.29466

9. Surveillance of cirrhosis for hepatocellular carcinoma: systematic review and economic analysis - PubMed [Internet]. [cited 2025 Jun 23]. Available from: https://pubmed.ncbi.nlm.nih.gov/17767898/

10. Impact of acute decompensation on the prognosis of patients with hepatocellular carcinoma | PLOS One [Internet]. [cited 2025 Jun 23]. Available from: https://journals.plos.org/plosone/article?id=10.1371/journal.pone.0261619

11. Yau T, Tang VYF, Yao TJ, Fan ST, Lo CM, Poon RTP. Development of Hong Kong Liver Cancer staging system with treatment stratification for patients with hepatocellular carcinoma. Gastroenterology. 2014 Jun;146(7):1691-1700.e3. doi:10.1053/j.gastro.2014.02.032 PubMed PMID: 24583061.

12. Legislative Council Secretariat. Health Services. 2021.

13. Zhao C, Jin M, Le RH, Le MH, Chen VL, Jin M, et al. Poor adherence to hepatocellular carcinoma surveillance: A systematic review and meta-analysis of a complex issue. Liver Int Off J Int Assoc Study Liver. 2018 Mar;38(3):503–14. doi:10.1111/liv.13555 PubMed PMID: 28834146.

14. Zhang M, Li Y, Fan Z, Shen D, Huang X, Yu Q, et al. Assessing health-related quality of life and health utilities in patients with chronic hepatitis B-related diseases in China: a cross-sectional study [Internet]. 2021 Sep 1. doi:10.1136/bmjopen-2020-047475

15. Yuen MF, Cheng CC, Lauder IJ, Lam SK, Ooi CG, Lai CL. Early detection of hepatocellular carcinoma increases the chance of treatment: Hong Kong experience. Hepatol Baltim Md. 2000 Feb;31(2):330–5. doi:10.1002/hep.510310211 PubMed PMID: 10655254.

16. Leung MK, Ko M, Chen J, Garay U, Leung B, Chow C, et al. Surveillance of Hepatocellular Cancer Among Hepatitis B and Cirrhosis Patients Using Protein Induced by Vitamin K Absence-II (PIVKA-II): A Cost-Utility Analysis for Hong Kong as an Example of Endemic Regions [Internet]. 2023 Mar 14 [cited 2025 Jun 23]. Available from: https://clinicalvalue.com/surveillance-of-hepatocellular-cancer-among-hepatitis-b-and-cirrhosis-patients-using-protein-induced-by-vitamin-k-absence-ii-pivka-ii-a-cost-utility-analysis-for-hong-kong-as-an-example-of-endemic/

17. Home Affairs Bureau. ITEM FOR PUBLIC WORKS SUBCOMMITTEE OF FINANCE COMMITTEE. 2008.

18. Census and Statistics Department. C&SD : Table 310-31001 : Gross Domestic Product (GDP), implicit price deflator of GDP and per capita GDP [Internet]. 2024 [cited 2025 Mar 20]. Available from: https://www.censtatd.gov.hk/en/web_table.html?id=310-31001

19. Tzartzeva K, Obi J, Rich NE, Parikh ND, Marrero JA, Yopp A, et al. Surveillance Imaging and Alpha Fetoprotein for Early Detection of Hepatocellular Carcinoma in Patients With Cirrhosis: A Meta-analysis. Gastroenterology. 2018 May;154(6):1706-1718.e1. doi:10.1053/j.gastro.2018.01.064 PubMed PMID: 29425931; PubMed Central PMCID: PMC5927818.

20. Elecsys PIVKA-II and Elecsys AFP assays demonstrate good clinical performance for hepatocellular carcinoma (HCC) diagnosis, across different disease stages and aetiologies [Internet]. [cited 2025 Jun 18]. Available from: https://medically.gene.com/global/en/unrestricted/oncology/diseases/gastrointestinal-cancer/materials/elecsys-pivka-ii-and-elecsys-afp-assays-demonstrate-goo.html

21. Pinjaroen N, Pan-Ngum W, Poovorawan K, Wastlund D, Mueller F, Lu P, et al. Economic evaluation of biomarker-based surveillance for Hepatocellular Carcinoma in Thai patients with Compensated Liver Cirrhosis [Internet]. doi:10.1371/journal.pone.0337913

22. Pham TTT, Ho DT, Nguyen TB, Phan HT. Clinical performance of GAAD score, alpha-fetoprotein, and PIVKA-II in diagnosing hepatocellular carcinoma in the Vietnamese cohort. Discov Oncol. 2025 Oct 3;16(1):1813. doi:10.1007/s12672-025-03651-4

23. Nan Y, Garay OU, Lu X, Zhang Y, Xie L, Niu Z, et al. Early-stage hepatocellular carcinoma screening in patients with chronic hepatitis B in China: a cost-effectiveness analysis. J Comp Eff Res. 2024 Apr;13(4):e230146. doi:10.57264/cer-2023-0146 PubMed PMID: 38415341; PubMed Central PMCID: PMC11044951.

24. D’Amico G, Garcia-Tsao G, Pagliaro L. Natural history and prognostic indicators of survival in cirrhosis: a systematic review of 118 studies. J Hepatol. 2006 Jan;44(1):217–31. doi:10.1016/j.jhep.2005.10.013 PubMed PMID: 16298014.

25. Trebicka J, Fernandez J, Papp M, Caraceni P, Laleman W, Gambino C, et al. The PREDICT study uncovers three clinical courses of acutely decompensated cirrhosis that have distinct pathophysiology. J Hepatol. 2020 Oct;73(4):842–54. doi:10.1016/j.jhep.2020.06.013 PubMed PMID: 32673741.

26. Khalaf N, Ying J, Mittal S, Temple S, Kanwal F, Davila J, et al. Natural History of Untreated Hepatocellular Carcinoma in a US Cohort and the Role of Cancer Surveillance. Clin Gastroenterol Hepatol Off Clin Pract J Am Gastroenterol Assoc. 2017 Feb;15(2):273-281.e1. doi:10.1016/j.cgh.2016.07.033 PubMed PMID: 27521507.

27. Yim SY, Seo YS, Jung CH, Kim TH, Lee JM, Kim ES, et al. The management and prognosis of patients with hepatocellular carcinoma: what has changed in 20 years? Liver Int Off J Int Assoc Study Liver. 2016 Mar;36(3):445–53. doi:10.1111/liv.12960 PubMed PMID: 26352789.

28. Fattovich G. Natural history of hepatitis B. J Hepatol. 2003 Jan 1;39:50–8. doi:10.1016/S0168-8278(03)00139-9
