# Supplementary material for: Surveillance of hepatocellular carcinoma (HCC) patients using Protein Induced by Vitamin K (PIVKA-II): A cost-utility analysis for Hong Kong
Source: PLoS One. 2026 Jul 17;21(7):e0353882. doi: 10.1371/journal.pone.0353882 (PMC13378965; doi:10.1371/journal.pone.0353882)
Supplement: S3 Appendix — (DOCX) [file pone.0353882.s003.docx]

# S3 Appendix: Extended results

Table 1: Breakdown of total costs per patient (HK $, undiscounted)

|  | **US + AFP** | **PIVKA-II + AFP** | **US (alone)** |
| --- | --- | --- | --- |
| Surveillance | 8,908 | 2,888 | 7,131 |
| HCC treatment A0 | 39,843 | 43,337 | 33,574 |
| HCC treatment BCD | 3,045 | 2,593 | 3,675 |
| FP | 20,980 | 19,471 | 10,498 |
| LC | 109,504 | 109,375 | 109,751 |
| DCLC | 109,414 | 109,414 | 109,414 |
| Palliative | 5,326 | 4,942 | 6,076 |
| TP | 1,021 | 1,074 | 901 |
| **Total costs (HK $)** | **298,923** | **294,004** | **281,813** |

Table 2: Diagnostic, cost, health and cost-effectiveness outcomes for bi-annual HCC surveillance methods in a CHB only population

Source:

|  | **US + AFP** | | **PIVKA-II + AFP** | | **US (alone)** | |
| --- | --- | --- | --- | --- | --- | --- |
| **Diagnostic outcomes (per 10,000 individuals enrolled in HCC surveillance)** | | | | | | |
| Total cases | 1,239 | | 1,239 | | 1,239 | |
| Detected early stage | 620 | | 674 | | 503 | |
| Detected late stage | 121 | | 97 | | 127 | |
| Detected incidentally | 49 | | 44 | | 65 | |
| Detected symptomatically | 449 | | 424 | | 544 | |
| % of early detection | 50.0% | | 54.4% | | 40.6% | |
| True positive | 741 | | 771 | | 630 | |
| True negative | 107,499 | | 108,942 | | 117,703 | |
| False negative | 233 | | 159 | | 439 | |
| False positive | 20,437 | | 16,994 | | 10,233 | |
| **Total cost and health outcomes (per individual)** | | | | | | |
| Total costs (HK $) | 211,344 | | 206,373 | | 199,110 | |
| Total life years | 12.0 | | 12.0 | | 11.9 | |
| Total QALYs | 9.06 | | 9.08 | | 9.02 | |
| **Cost-effectiveness (per individual)** | | | | | | |
|  | | **Incremental costs (HK $)** | | **Incremental QALYs** | | **ICER per QALY gained (HK $)** |
| ‘PIVKA-II + AFP’ vs. ‘US + AFP’ | | -4,971 | | 0.013 | | Dominant |
| ‘PIVKA-II + AFP’ vs. US (alone) | | 7,264 | | 0.052 | | 139,310 |

Abbreviations: AFP: alpha-fetoprotein; CHB: chronic hepatitis B; HCC: hepatocellular carcinoma; ICER: incremental cost-effectiveness ratio; PIVKA-II: protein induced by vitamin K absence or antagonist-II; US: ultrasound.

Note: Results for CHB population only were based upon 10,000 microsimulations and hence differ slightly from those of the main analysis (n=50,000).

Table 3: Diagnostic, cost, health and cost-effectiveness outcomes for bi-annual HCC surveillance methods in a LC only population

|  | **US + AFP** | | **PIVKA-II + AFP** | | **US (alone)** | |
| --- | --- | --- | --- | --- | --- | --- |
| **Diagnostic outcomes (per 10,000 individuals enrolled in HCC surveillance)** | | | | | | |
| Total cases | 1,838 | | 1,838 | | 1,838 | |
| Detected early stage | 919 | | 1,047 | | 723 | |
| Detected late stage | 167 | | 132 | | 204 | |
| Detected incidentally | 63 | | 56 | | 88 | |
| Detected symptomatically | 688 | | 602 | | 822 | |
| % of early detection | 50.0% | | 57.0% | | 39.4% | |
| True positive | 1,086 | | 1,179 | | 927 | |
| True negative | 70,363 | | 63,479 | | 77,080 | |
| False negative | 477 | | 283 | | 806 | |
| False positive | 13,539 | | 20,423 | | 6,822 | |
| **Total cost and health outcomes (per individual)** | | | | | | |
| Total costs (HK $) | £343,865 | | £348,067 | | £332,228 | |
| Total life years | 8.9 | | 9.0 | | 8.9 | |
| Total QALYs | 6.55 | | 6.59 | | 6.49 | |
| **Cost-effectiveness (per individual)** | | | | | | |
|  | | **Incremental costs (HK $)** | | **Incremental QALYs** | | **ICER per QALY gained (HK $)** |
| ‘PIVKA-II + AFP’ vs. ‘US + AFP’ | | 4,202 | | 0.037 | | 112,386 |
| ‘PIVKA-II + AFP’ vs. US (alone) | | 15,839 | | 0.0096 | | 165,217 |

Abbreviations: AFP: alpha-fetoprotein; LC: Liver cirrhosis; B; HCC: hepatocellular carcinoma; ICER: incremental cost-effectiveness ratio; PIVKA-II: protein induced by vitamin K absence or antagonist-II; US: ultrasound.

Note: Results for LC population only were based upon 10,000 microsimulations and hence differ slightly from those of the main analysis (n=50,000).
