# Supplementary material for: Surveillance of hepatocellular carcinoma (HCC) patients using Protein Induced by Vitamin K (PIVKA-II): A cost-utility analysis for Hong Kong
Source: PLoS One. 2026 Jul 17;21(7):e0353882. doi: 10.1371/journal.pone.0353882 (PMC13378965; doi:10.1371/journal.pone.0353882)
Supplement: S4 Appendix — (DOCX) [file pone.0353882.s004.docx]

# S4 Appendix: Sensitivity analyses

One-way sensitivity analysis for net monetary benefit (NMB) was performed by varying the cost, diagnostic performance, utility, and transition probabilities. NMB is closely linked to the ICER per QALY gained, but mathematically easier to interpret since negative results are not possible. The sensitivity analysis results for the comparison between ‘PIVKA+AFP’ vs ‘US + AFP’ are presented in Figure 1. The analysis shows that the diagnostic accuracy of ‘US + AFP’, HCC and DCLC incidence, and the cost of the screening tests and false positive diagnoses were among the most impactful factors for the model’s cost-effectiveness results. The relatively low variability of NMB suggests that the conclusions from this analysis are quite robust towards changes in individual model parameters.

Figure 1: One-way sensitivity analysis results for the comparison between PIVKA-II+AFP vs ‘US + AFP’


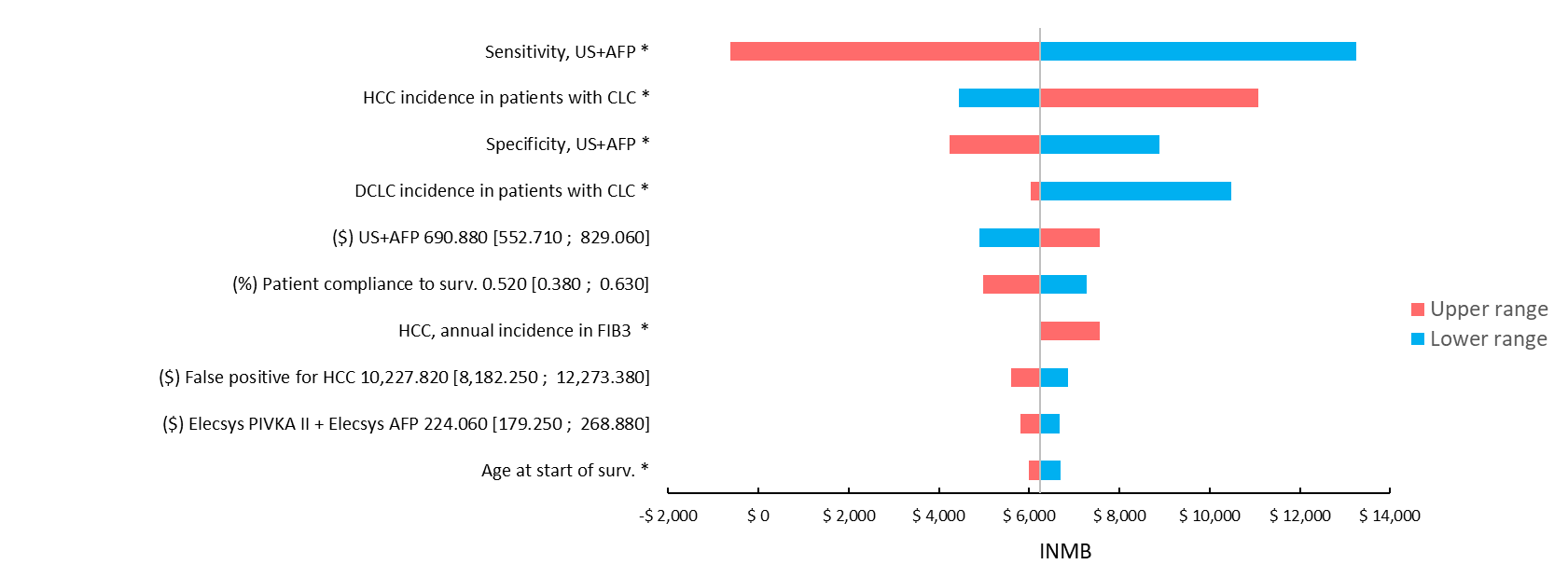


Probabilistic sensitivity analysis (PSA) was conducted by simultaneously varying all model input parameters through Monte-Carlo analysis. The results are summarized in a cost-effectiveness plane in Figure 2. Compared to ‘US + AFP’, all the simulations for ‘PIVKA-II + AFP’ resulted in both QALY gain and cost reductions. This suggests that ‘PIVKA-II + AFP would be a dominant surveillance strategy from a cost-effectiveness standpoint, and that results are relatively robust to uncertainty in the data used for the analysis.

Figure 2: Cost-effectiveness plane for HCC surveillance using PIVKA-II and PIVKA-II+AFP versus US (alone) or US + AFP


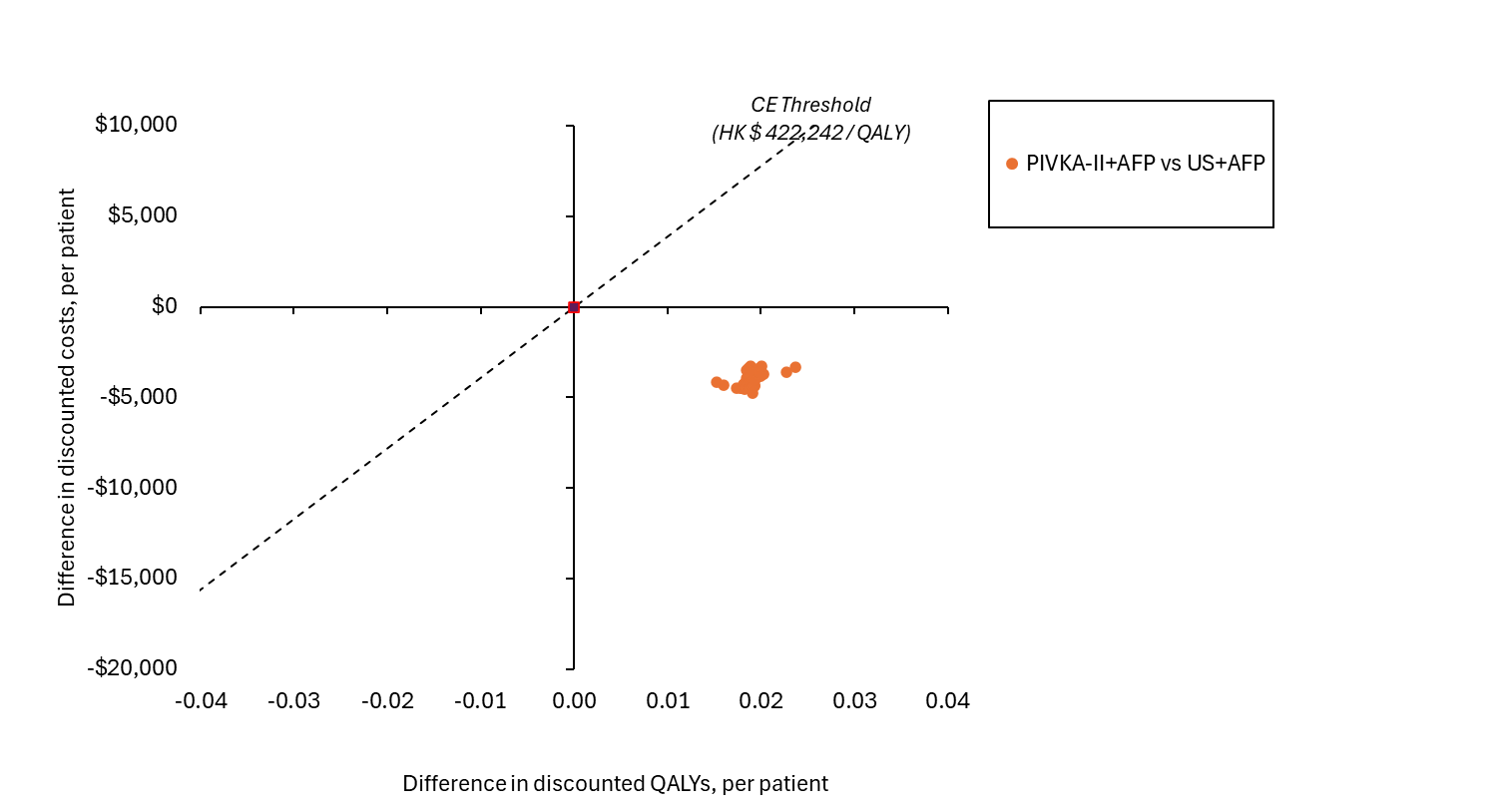


The scenario analyses were selected based on clinical experience and previously published literature or identified as impactful parameters for the model’s results. Scenarios included:

- Surveillance age between 40-50 years across all aetiologies
- Surveillance age between 50-60 years across all aetiologies
- Surveillance age between 60-70 years across all aetiologies
- Discount rate of 0% (for both costs and QALYs)
- Discount rate of 4.0% (for both costs and QALYs)
- 100% compliance rate for all methods
- 75% compliance rate for all methods
- Surveillance specific compliance rates (75% compliance rate for PIVKA-II based assessment vs. base case rates for ultrasound-based assessment)
- Alternative data on diagnostic accuracy for ‘PIVKA-II + AFP’, based upon Nan et al. (2024): early-stage sensitivity: 76.1%, all-stage sensitivity: 85.3%, specificity: 90.4%
- Alternative costs for ultrasound: HK $ 1,000 for US (alone) and HK $ 1,138 for US + AFP
- Alternative cost for PIVKA-II + AFP: HK $ 448.13 (twice that of the base case)
- Alternative cost of confirmatory testing: The cost of confirmatory testing for suspected HCC positive patients (both true and false positives) are reduced by half

For computational efficiency, each scenario analysis was modelled using 10,000 microsimulations, as opposed to 50,000 for the base case. For this reason, results from the scenario analyses deviated slightly from those of the base case, even if all other model settings were identical.

Results for total costs and QALYs from the scenario analyses for all surveillance methods are presented in the table below. ICERs per QALY were compared against ‘US + AFP’ since this is the recommended standard-of-care. These scenario analyses showed that although the overall level of costs and QALYs may vary substantially between scenarios, the relative cost-effectiveness of the different surveillance methods remained relatively stable. The combination of PIVKA-II plus AFP was the preferred method for HCC surveillance from a cost-effectiveness perspective across all scenarios. Overall, the scenarios with the greatest impact upon the relative cost-effectiveness of the different strategies were the prices for ultrasound and PIVKA-II, as well as when assuming perfect patient compliance to surveillance.

Table 1: Scenario analyses, showing costs (HK$) and health outcomes per screened individual

|  | **US + AFP** | **PIVKA-II + AFP** | **US** |
| --- | --- | --- | --- |
| **Base case †** | | | |
| Total cost (HK $) | 208,823 | 204,635 | 195,879 |
| Total QALYs | 8.991 | 9.008 | 8.961 |
| ICER vs. US + AFP (HK $) | N/A | Dominant | Loss of QALYs (430,212) |
| **Surveillance age limit: 40-50 years** | | | |
| Total cost (HK $) | 198,979 | 196,576 | 190,019 |
| Total QALYs | 9.081 | 9.094 | 9.057 |
| ICER vs. US + AFP (HK $) | N/A | Dominant | Loss of QALYs (378,526) |
| **Surveillance age limit: 50-60 years** | | | |
| Total cost (HK $) | 195,405 | 192,981 | 186,539 |
| Total QALYs | 8.755 | 8.767 | 8.733 |
| ICER vs. US + AFP (HK $) | N/A | Dominant | Loss of QALYs (404,644) |
| **Surveillance age limit: 60-70 years** | | | |
| Total cost (HK $) | 194,244 | 191,893 | 185,406 |
| Total QALYs | 8.581 | 8.594 | 8.560 |
| ICER vs. US + AFP (HK $) | N/A | Dominant | Loss of QALYs (418,794) |
| **Discount rate: 0% for costs and QALYs** | | | |
| Total cost (HK $) | 297,024 | 292,048 | 278,207 |
| Total QALYs | 13.093 | 13.121 | 13.036 |
| ICER vs. US + AFP (HK $) | N/A | Dominant | Loss of QALYs (334,167) |
| **Discount rate: 4.0% for costs and QALYs** | | | |
| Total cost (HK $) | 189,593 | 185,655 | 177,916 |
| Total QALYs | 8.131 | 8.145 | 8.106 |
| ICER vs. US + AFP (HK $) | N/A | Dominant | Loss of QALYs (463,948) |
| **Surveillance compliance rate: 100%** | | | |
| Total cost (HK $) | 236,814 | 227,778 | 215,393 |
| Total QALYs | 9.100 | 9.126 | 9.061 |
| ICER vs. US + AFP (HK $) | N/A | Dominant | Loss of QALYs (552,070) |
| **Surveillance compliance rate: 75%** | | | |
| Total cost (HK $) | 223,841 | 217,472 | 206,119 |
| Total QALYs | 9.059 | 9.082 | 9.019 |
| ICER vs. US + AFP (HK $) | N/A | Dominant | Loss of QALYs (440,631) |
| **Surveillance compliance rate: 75% for PIVKA-II, 52% for ultrasound** | | | |
| Total cost (HK $) | 195,879 | 204,479 | 208,823 |
| Total QALYs | 8.961 | 9.047 | 8.991 |
| ICER vs. US + AFP (HK $) | N/A | ICER: 100,539 | ICER: 430,212 |
| **Alternative diagnostic accuracy for 'PIVKA-II + AFP' (Nan et al., 2024)** | | | |
| Total cost (HK $) | 208,823 | 204,754 | 195,879 |
| Total QALYs | 8.991 | 9.008 | 8.961 |
| ICER vs. US + AFP (HK $) | N/A | Dominant | Loss of QALYs (430,212) |
| **Alternative ultrasound cost: HK $ 1,000 for US (alone), HK $ 1,138 for ‘US + AFP’** | | | |
| Total cost (HK $) | 213,313 | 204,635 | 200,372 |
| Total QALYs | 8.991 | 9.008 | 8.961 |
| ICER vs. US + AFP (HK $) | N/A | Dominant | Loss of QALYs (430,109) |
| **PIVKA-II costs twice as high** | | | |
| Total cost (HK $) | 208,823 | 206,883 | 195,879 |
| Total QALYs | 8.991 | 9.008 | 8.961 |
| ICER vs. US + AFP (HK $) | N/A | Dominant | Loss of QALYs (430,212) |
| **Confirmatory testing costs reduced by half** | | | |
| Total cost (HK $) | 200,703 | 197,023 | 191,828 |
| Total QALYs | 8.991 | 9.008 | 8.961 |
| ICER vs. US + AFP (HK $) | N/A | Dominant | Loss of QALYs (294,984) |

† Base case results for scenario analysis were based upon 10,000 microsimulations and hence differ slightly from those of the main analysis (n=50,000).
