# Supplementary material for: Surveillance of hepatocellular carcinoma (HCC) patients using Protein Induced by Vitamin K (PIVKA-II): A cost-utility analysis for Hong Kong
Source: PLoS One. 2026 Jul 17;21(7):e0353882. doi: 10.1371/journal.pone.0353882 (PMC13378965; doi:10.1371/journal.pone.0353882)
Supplement: Attachment — Submitted filename: Response to Reviewers v3.0 20260331.docx [file pone.0353882.s007.docx]

PONE-D-25-51132: Response to Reviewers

**Manuscript ID:** PONE-D-25-51132

**Revision #:** 1

**Title:** Surveillance of hepatocellular carcinoma (HCC) patients using Protein Induced by Vitamin K (PIVKA-II): A cost-utility analysis for Hong Kong

**Journal:** PLOS ONE

## Document information

We have previously submitted a manuscript to PLOS ONE. The journal has now provided feedback to this manuscript, including comments following their peer-review process. This document contains all feedback we have received from the journal, as well as our responses to this feedback.

## Editorial comments and author responses

When submitting your revision, we need you to address these additional requirements.

1. Please ensure that your manuscript meets PLOS ONE's style requirements, including those for file naming. The PLOS ONE style templates can be found at

<https://journals.plos.org/plosone/s/file?id=wjVg/PLOSOne_formatting_sample_main_body.pdf> and

<https://journals.plos.org/plosone/s/file?id=ba62/PLOSOne_formatting_sample_title_authors_affiliations.pdf>

Author response A-1:

We have formatted the updated manuscript to align with the journal requirements listed above.

2. Please note that PLOS One has specific guidelines on code sharing for submissions in which author-generated code underpins the findings in the manuscript. In these cases, we expect all author-generated code to be made available without restrictions upon publication of the work. Please review our guidelines at https://journals.plos.org/plosone/s/materials-and-software-sharing#loc-sharing-code and ensure that your code is shared in a way that follows best practice and facilitates reproducibility and reuse.

Author response A-2:

The economic model was developed in Microsoft Excel. We have provided an updated Data Availability Statement to describe how readers can access the model if needed.

3. Please update your submission to use the PLOS LaTeX template. The template and more information on our requirements for LaTeX submissions can be found at <http://journals.plos.org/plosone/s/latex>

Author response A-3:

We have prepared the manuscript formatting in line with PLOS ONE’s author guidelines. If any further edits are required, such as adapting the LaTeX template, we are happy to accommodate them.

4. We note that the grant information you provided in the ‘Funding Information’ and ‘Financial Disclosure’ sections do not match.

When you resubmit, please ensure that you provide the correct grant numbers for the awards you received for your study in the ‘Funding Information’ section.

Author response A-4:

We have now moved our statement on funding to the “Financial disclosure” section. No grant numbers are applicable.

5. Thank you for stating the following financial disclosure:

“The health economic model used for this analysis was funded by Roche Diagnostics Asia Pacific Ltd, which also provided access to data on diagnostic accuracy. QZ and JC are previous employees of Roche Diagnostics. DW and TT are consultants at Vista Health, a company that have received funding from Roche Diagnostics for evaluating the cost-effectiveness of alternative HCC surveillance methods. MFY has received compensation at fair market value for his consultancy in model advisory boards of Roche Diagnostics and for delivering lectures organised by Roche Diagnostics. The opinions expressed in this publication are those of the authors and they do not necessarily reflect the view of the funder.”

Please state what role the funders took in the study. If the funders had no role, please state: "The funders had no role in study design, data collection and analysis, decision to publish, or preparation of the manuscript."

If this statement is not correct you must amend it as needed.

Please include this amended Role of Funder statement in your cover letter; we will change the online submission form on your behalf.

Author response A-5:

We have prepared an updated financial disclosure statement which better reflects the role of the funder. We have submitted this within the cover letter of our revised manuscript.

6. Thank you for stating the following financial disclosure:

“The health economic model used for this analysis was funded by Roche Diagnostics Asia Pacific Ltd, which also provided access to data on diagnostic accuracy. QZ and JC are previous employees of Roche Diagnostics. DW and TT are consultants at Vista Health, a company that have received funding from Roche Diagnostics for evaluating the cost-effectiveness of alternative HCC surveillance methods. MFY has received compensation at fair market value for his consultancy in model advisory boards of Roche Diagnostics and for delivering lectures organised by Roche Diagnostics. The opinions expressed in this publication are those of the authors and they do not necessarily reflect the view of the funder.”

We note that one or more of the authors is affiliated with the funding organization, indicating the funder may have had some role in the design, data collection, analysis or preparation of your manuscript for publication; in other words, the funder played an indirect role through the participation of the co-authors. If the funding organization did not play a role in the study design, data collection and analysis, decision to publish, or preparation of the manuscript and only provided financial support in the form of authors' salaries and/or research materials, please do the following:

1. Review your statements relating to the author contributions, and ensure you have specifically and accurately indicated the role(s) that these authors had in your study. These amendments should be made in the online form.

2. Confirm in your cover letter that you agree with the following statement, and we will change the online submission form on your behalf:

“The funder provided support in the form of salaries for authors [insert relevant initials], but did not have any additional role in the study design, data collection and analysis, decision to publish, or preparation of the manuscript. The specific roles of these authors are articulated in the ‘author contributions’ section.

Author response A-6:

We have prepared an updated Funding statement for this manuscript which is submitted alongside the revised manuscript.

7. In the online submission form, you indicated that “All data used for this analysis are included in the article. In most cases, data have been obtained from other published studies, and these are referenced within the manuscript.

All supporting calculations and analyses can be made available upon reasonable request to the corresponding author. “

All PLOS journals now require all data underlying the findings described in their manuscript to be freely available to other researchers, either 1. In a public repository, 2. Within the manuscript itself, or 3. Uploaded as supplementary information.

This policy applies to all data except where public deposition would breach compliance with the protocol approved by your research ethics board. If your data cannot be made publicly available for ethical or legal reasons (e.g., public availability would compromise patient privacy), please explain your reasons on resubmission and your exemption request will be escalated for approval.

Author response A-7:

We have updated our Data Availability Statement (see below). All data used for the analysis is either found within the main manuscript or as supplementary information (Appendix S2 contain a comprehensive list of model data inputs).

"All data used for this analysis are included in the article. In most cases, data have been obtained from other published studies, and these are referenced within the manuscript. All supporting calculations and analyses can be made available upon reasonable request to the corresponding author"

8. If the reviewer comments include a recommendation to cite specific previously published works, please review and evaluate these publications to determine whether they are relevant and should be cited. There is no requirement to cite these works unless the editor has indicated otherwise.

Author response A-8:

In response to some reviewer comments, we have updated the manuscript with additional information and corresponding sources. These are all listed in the updated list of references.

[Note: HTML markup is below. Please do not edit.]

## Reviewer's Responses to Questions

Comments to the Author

**1. Is the manuscript technically sound, and do the data support the conclusions?**

The manuscript must describe a technically sound piece of scientific research with data that supports the conclusions. Experiments must have been conducted rigorously, with appropriate controls, replication, and sample sizes. The conclusions must be drawn appropriately based on the data presented.

**Reviewer #1:** Yes

**Reviewer #2:** Yes

**Reviewer #3:** Partly

**2. Has the statistical analysis been performed appropriately and rigorously?**

**Reviewer #1:** Yes

**Reviewer #2:** Yes

**Reviewer #3:** Yes

**3. Have the authors made all data underlying the findings in their manuscript fully available?**

The PLOS Data policy requires authors to make all data underlying the findings described in their manuscript fully available without restriction, with rare exception (please refer to the Data Availability Statement in the manuscript PDF file). The data should be provided as part of the manuscript or its supporting information, or deposited to a public repository. For example, in addition to summary statistics, the data points behind means, medians and variance measures should be available. If there are restrictions on publicly sharing data—e.g. participant privacy or use of data from a third party—those must be specified.

**Reviewer #1:** Yes

**Reviewer #2:** Yes

**Reviewer #3:** Yes

**4. Is the manuscript presented in an intelligible fashion and written in standard English?**

PLOS ONE does not copyedit accepted manuscripts, so the language in submitted articles must be clear, correct, and unambiguous. Any typographical or grammatical errors should be corrected at revision, so please note any specific errors here.

**Reviewer #1:** Yes

**Reviewer #2:** Yes

**Reviewer #3:** Yes

## Review Comments to the Author

Please use the space provided to explain your answers to the questions above. You may also include additional comments for the author, including concerns about dual publication, research ethics, or publication ethics. (Please upload your review as an attachment if it exceeds 20,000 characters)

### **Reviewer #1:**

The manuscript is well written, clearly structured, and addresses an important and timely topic in hepatocellular carcinoma (HCC) surveillance. The authors present a cost-utility analysis comparing “PIVKA-II + AFP” with the current standard of care “US + AFP” in Hong Kong, using a Markov microsimulation model.

The study is relevant, methodologically sound, and offers valuable insights into health economic decision-making and screening policy in a high-burden region. Overall, it provides a meaningful contribution to the field. However, a number of points should be considered to further strengthen the manuscript.

• The abstract is informative, but it could briefly clarify that the study uses a model-based economic evaluation relying on published data rather than primary patient-level data.

Author response B-1:

We have now updated the abstract accordingly.

• The introduction provides good background but would benefit from a clearer statement of novelty—what this study adds beyond previous cost-effectiveness studies of biomarker-based HCC surveillance in Asia.

Author response B-2:

We’ve added a sentence to the introduction to highlight that the cost-effectiveness of ‘PIVKA-II + AFP’ has not previously been evaluated in Asia Pacific.

• The rationale for focusing on Hong Kong is well justified; however, the authors could discuss the potential generalizability of their findings to other similar health systems or regions with limited ultrasound capacity.

Author response B-3:

We have now added some thoughts about the generalisability for our findings for other healthcare systems in our Discussion. We believe some of the key findings regarding the potential reduction of false positive diagnoses and the scalability of biomarker-based screening may be valuable to similar health systems and regions. However, we recommend that the value of PIVKA-II-based screening is assessed in those settings prior to policy changes, since cost-effectiveness may differ between different healthcare settings.

• The Methods section is comprehensive, but additional details on model validation, calibration, and verification by local experts would improve transparency. It would be useful to mention whether the model was externally validated or cross-checked with real-world data.

Author response B-4:

We agree that further validation would be valuable. We have compared our results to those from a similar recent model-based cost-effectiveness analysis from mainland China, by Nan et al. (2024). That study focused solely on non-cirrhotic patients with chronic hepatitis B, but overall the results were similar once adjusted for patient characteristics.

For validation purposes, it is important that clinical outcomes are distinguished by tumour stage, since the purpose of routine HCC surveillance in not only to identify HCC cases, but to identify them sufficiently early to initiate effective treatment. To the best of our knowledge, the only larger clinical study which have published HCC outcomes in Hong Kong across different HCC stages is the study by Yau et al. (2014), which was used to derive the survival curves for this economic analysis.

• The choice of the Markov microsimulation model is appropriate, yet a schematic justification for the health states and transition logic (e.g., CHB → LC → HCC stages) should be presented in clearer terms for readers unfamiliar with economic modeling.

Author response B-5:

We thank the reviewer for this suggestion. We have now added a reference to the meta-analysis on cost-effectiveness evaluation of HCC surveillance by Nguyen et al. (2021).

For readers wanting even more details, we describe the framework in both Figure 1 and Appendix S. For even further information, the model framework has been previously explained by Garay et al. (2024), which is referenced within our manuscript.

• The derivation of QALYs should be described in greater detail, including how utility values were obtained, whether local Hong Kong data were available, and how these values were applied across disease states.

Author response B-6:

We agree with the reviewer and have updated the manuscript accordingly. Further, we have managed to identified data from Zhang et al. (2021) for utility values derived from 1,071 Chinese patients with chronic Hepatitis B, CLC, DCLC and different stages of HCC. We have updated the analysis to use these data instead, since 1) this is more applicable for our target population, and 2) this data contained utility values for all stages, decreasing the risk of biasing the analysis by using utility values from multiple sources.

• Transition probabilities and epidemiological parameters appear to be drawn from a mixture of local and international sources; the paper should clarify how these were harmonized or adapted to Hong Kong’s context.

Author response B-7:

We have now added a paragraph to the manuscript about how suitable data for Hong Kong was identified and selected.

• The time horizon and discount rate (3%) are standard; however, the results would be more robust if sensitivity analyses around these parameters were presented explicitly.

Author response B-8:

The results from two scenarios using alternative discount rates (0% and 4.0% for both cost and QALYs) are presented in Appendix S4.

Our scenario analyses shows that whilst the discount rates have a big impact upon the overall costs and QALYs, varying these have minor impact upon the *relative* value of the different screening methods compared to one another.

• Cost inputs from older studies (e.g., Yuen et al. 2000) should either be updated or clearly adjusted for inflation to reflect current prices. The costing year should be explicitly stated.

Author response B-9:

Thank you for identifying this oversight. We have now updated all costs to a common price level, using the cost year 2024, and have added the source of the inflation index to the manuscript.

• The analysis correctly adopts a health system payer perspective, but the exclusion of indirect or societal costs (e.g., productivity loss) should be noted and justified.

Author response B-10:

We have added a paragraph in the manuscripts Discussion to address this. The absence of Hong Kong data on wider societal costs associated with HCC surveillance and HCC development/treatment meant that we had to use a more confined healthcare payer perspective. This is arguably the most important for policy considerations, but we believe that the benefit from a biomarker-based approach would be even greater if patients’ time and costs for screening and confirmatory testing could have been accounted for.

• The incremental cost-effectiveness ratio (ICER) results are well calculated, but the authors could improve presentation by including the base-case ICERs and the variation observed across sensitivity scenarios directly in the Results section.

Author response B-11:

We thank for the suggestion that we would usually agree with. However, in this specific case, most of the ICERs for the main comparison (‘PIVKA-II + AFP’ vs ‘US + AFP’) just reads as “Dominant”. We feel that presenting this information without also presenting the incremental costs and QALYs would not be very meaningful to the reader, but the latter approach would require a big table, especially if also considering a screening strategy of US (alone). For this reason, we suggest keeping all scenario results in Appendix S4, so as to not decrease the article’s readability.

• A cost-effectiveness acceptability curve (CEAC) or scatter plot from the probabilistic sensitivity analysis would help readers visually assess the uncertainty around cost-effectiveness.

Author response B-12:

A scatter plot showing the cost-effectiveness plane is presented in Appendix 4.

Since the main comparison (‘PIVKA-II + AFP’ vs ‘US + AFP’) results in the standard-of-care being dominated, we feel that a cost-effectiveness acceptability curve (CEAC) would offer limited value to the reader.

• The chosen cost-effectiveness threshold (GDP per capita of HK$422,242 per QALY) is reasonable, but the discussion should acknowledge that there is no official threshold in Hong Kong and explore how results might differ under alternative thresholds.

Author response B-13:

We have clarified in the manuscript that Hong Klong does not have an official threshold for cost-effectiveness.

Since ‘PIVKA-II + AFP’ is dominant – i.e. both improving health and lowering costs – versus the recommended standard-of-care (‘US + AFP’), using an alternative threshold for cost-effectiveness would not change our conclusions. For this reason, we have not expanded the manuscript with a cost-effectiveness acceptability curve (CEAC) or a discussion about the impact of Hong Kong’s cost-effectiveness threshold.

• The financial disclosure statement indicates strong involvement of Roche Diagnostics, including data provision and consultancy relationships. This potential source of bias should be discussed more openly within the limitations section.

Author response B-14:

We have updated the submission with an updated statement on funding information and financial disclosure, on top of the submitted conflict of interest disclosures. The journal will make these available to all readers. To not decrease the manuscript’s readability, we prefer to discuss this potential source of bias in these statements rather than within the main text.

• Some diagnostic accuracy data are listed as “Roche diagnostics, data on file.” The authors should clarify whether these values have been independently validated or published in peer-reviewed form.

Author response B-15:

We have now updated the analysis to use diagnostic accuracy data from already published sources, instead of the unpublished data from Roche used for the original submission. All data are now presented within the main manuscript or the supporting documents.

• The Results section is detailed and logically organized, but Table 2 could be simplified or moved in part to supplementary materials to improve readability.

Author response B-16:

Due to their importance to our findings and the ensuing discussion, we prefer to present these results at their current place in Table 2.

Though we would prefer to leave as it currently is, we defer to PLOS ONE to decide whether Table 2 should be condensed further, since they need to consider the manuscript’s layout. If PLOS ONE wants to condense Table 2 further we believe there are two ways to do this while retaining the most crucial information:

1) The 4 rows showing results for “Detected early stage”, “Detected late stage”, “Detected incidentally”, “Detected symptomatically” could be moved to appendix S3, since these can already largely be inferred from table 2 by “% of early detection”.

2) The column for “US (alone)” could be moved to appendix S3, as the other two comparators are the main ones for the analysis.

• Subgroup and sensitivity analyses are well conceived, though a clearer explanation of the drivers of cost-effectiveness between cirrhotic and non-cirrhotic groups would help interpretation.

Author response B-17:

The driver of cost-effectiveness for cirrhotic vs non-cirrhotic patients come down to the different diagnostic accuracy data observed for these groups. This in turns stems largely from the different levels of AFP in the serum. The presence of HCC tumours greatly increases AFP-levels, which makes AFP a useful biomarker for detecting the presence of HCC. However, liver cirrhosis also increases AFP level. Within non-cirrhotic patients, the risk of obtaining a false positive diagnosis when using either ‘US + AFP’ or ‘PIVKA-II + AFP’ is low. However, this increases greatly among cirrhotic patients, with an increased risk of unwarranted medical expenses due to unnecessary confirmatory testing as a result. We have added a brief explanation of this to the manuscript.

• The discussion effectively summarizes the main findings but should include a stronger acknowledgment that model-based results depend on assumptions and are not a substitute for real-world clinical evidence.

Author response B-18:

We have made acknowledgements throughout the manuscript that modelling relies upon assumptions and that policy should consider broader evidence than just the pieces we are providing. We’ve also updated the following statement within the Discussion:

“The results of this study should be interpreted with caution given the nature of the modelling methodology and assumptions. The results and should be carefully considered in context of the prevailing body of evidence and clinical opinion.”

• The policy implications could be highlighted more strongly—specifically, how biomarker-based surveillance might complement, rather than replace, ultrasound within Hong Kong’s HCC screening framework.

Author response B-19:

The manuscript discusses in several sections that there is currently a big discrepancy between the HCC surveillance that Hong Kong high-risk patients ought to get according to guidelines and the surveillance they actually receive. Access to ultrasound assessment is frequently cited as a bottleneck for HCC surveillance.

Routine HCC surveillance is repeated across several cycles among high-risk patients. In clinical practice, many patients may involuntarily skip screening cycles when they are not summoned for screening due to lack of available ultrasonographers. For such patients, screening using ‘PIVKA-II + AFP’ would constitute a feasible alternative for screening cycles where ultrasound plus AFP would not be offered. Many Hong Kong hospitals already have the processing equipment for HCC testing using ‘PIVKA-II + AFP’.

It has also been discussed by e.g. Lui et al. (2024) that for biomarker-based HCC surveillance using PIVKA-II could be useful in prioritising patient referrals to the private sector for imaging.

We want to point out again that we do not suggest that ‘PIVKA-II + AFP’ should replace ultrasound-based HCC surveillance in Hong Kong. However, the diagnostic performance appears sufficiently good that this would serve as a valuable supplement for patients in screening cycles where ultrasound-based screening would not be feasible.

• The conclusion could be shortened and focused more on the practical implications rather than restating quantitative findings already presented in the Results section.

Author response B-20:

We have edited the final paragraph to focus more on policy implications from our findings, rather than restating the quantitative results.

• The limitations section should emphasize potential uncertainty due to parameter estimates derived from literature and manufacturer data, and discuss the absence of head-to-head comparative trials.

Author response B-21:

We agree that the absence of head-to-head comparative trials for the diagnostic methods is a big limitation of this study. Within the manuscript we already state that:
“A limitation of this study is the absence of head-to-head comparison between the PIVKA-II-based and the US-based methods [… and] biases related to differences in study settings cannot be ruled out.”

Later in the Discussion, we also recommend that:
“Future studies should consider collecting head-to-head comparison data between PIVKA-II-based and US-based HCC surveillance methods, particularly in early-stage HCC detection, to provide more robust evidence.”

We have further expanded the discussion on the absence of head-to-head comparative trials and the potential biases that could arise from this. Finally, we have also added an additional scenario analysis using alternative data on diagnostic performance from Nan et al. to confirm the robustness of our findings in the light of uncertainty of the data on diagnostic performance.

• The ethical statement reads “N/A.” The authors may clarify why ethical approval was not applicable, given that secondary data sources were used.

Author response B-22:

We have now updated he section for “Ethics Statement” with the following information:
“*Ethics approval was not applicable since this cost-effectiveness analysis is based upon secondary data and no patient data was collected for this study*”

• Figures and tables should use consistent decimal places, abbreviations, and units. Minor language edits would further improve readability and flow.

Author response B-23:

We have updated all figures and tables to consistent decimal places (depending on the outcome measured), abbreviations and units. We have also provided some minor language improvement for increased readability.

• The data availability statement should ensure compliance with PLOS ONE’s open-data policy by specifying where model inputs and simulation codes can be accessed.

Author response B-24:

We have extended Appendix S2 to report all model parameters. Since the model was developed in Microsoft Excel it is not easy to share all simulation codes, however, the model can be shared upon reasonable request to the corresponding author.

• Future studies could explore real-world comparative data between “PIVKA-II + AFP” and “US + AFP,” potentially including combinations with ultrasound or other biomarkers to confirm the model’s assumptions.

Author response B-25:

We state in the manuscript that:
“Future studies should consider collecting head-to-head comparison data between PIVKA-II-based and US-based HCC surveillance methods, particularly in early-stage HCC detection, to provide more robust evidence. It should also evaluate the performance of combining PIVKA-II and AFP with ultrasound, as well as whether other biomarkers or combinations of biomarkers could provide even better diagnostic accuracy.”

### **Reviewer #2:**

Overall Assessment

This manuscript presents a well-structured and timely cost-utility analysis evaluating the use of PIVKA-II combined with AFP for HCC surveillance in Hong Kong. The authors adapt a Markov microsimulation model to local epidemiological and economic conditions, providing valuable insight into the potential role of biomarker-based surveillance in resource-limited settings. The study addresses an important gap in liver cancer screening policy and is highly relevant to clinical and public health decision-making in regions with high HBV prevalence.

The manuscript is generally well written, clearly structured, and appropriately referenced. The modelling framework, assumptions, and results are presented with transparency, and the conclusions are well aligned with the findings. Some of the major strengths of this manuscript have relevance and policy impact, methodological Transparency, comprehensive sensitivity analysis and balanced discussion. However, some clarifications and improvements would enhance the scientific rigour and reproducibility of the study.

**Major Comments**

**Model Validation and Calibration:**

Although the model is adapted from prior work, it would be helpful to describe how the model was validated or calibrated against observed Hong Kong data (e.g., incidence, stage distribution, or survival outcomes).

A visual comparison between simulated and empirical HCC detection rates or survival curves could strengthen confidence in model accuracy.

Author response C-1:

We agree that further validation would be valuable. We have compared our results to those from a similar recent model-based cost-effectiveness analysis from mainland China, by Nan et al. (2024). That study focused solely on non-cirrhotic patients with chronic hepatitis B, but overall the results were similar once adjusted for patient characteristics.

For validation purposes, it is important that clinical outcomes are distinguished by tumour stage, since the purpose of routine HCC surveillance in not only to identify HCC cases, but to identify them sufficiently early to initiate effective treatment. To the best of our knowledge, the only larger clinical study which have published HCC outcomes in Hong Kong across different HCC stages is the study by Yau et al. (2014). Since this study was used to derive the survival curves for this economic analysis, we felt that a visual comparison between the two would be of limited value to the reader.

**Perspective and Cost Components:**

The analysis adopts a healthcare system (payer) perspective. However, some key cost categories such as patient time costs or indirect costs are not discussed. Clarifying whether these were intentionally excluded and their potential influence on results would be valuable.

Please specify whether costs are adjusted to a reference year (e.g., 2024 HK$) and describe the inflation or exchange rate method used.

Author response C-2:

Due to the absence on Hong Kong data for patients’ costs associated with screening and HCC treatment, a more narrow healthcare payer perspective was used for this analysis. We have added a paragraph to the Discussion about how ignoring such indirect costs may impact our results. Compared to the currently recommended standard-of-care (‘US + AFP’), ‘PIVKA-II + AFP’ reduces the number of confirmatory testing following false positive diagnosis. It is therefore likely that the benefits from a biomarker-based approach would be greater if patients’ time and costs for screening and confirmatory testing could have been accounted for.

We have now adjusted all costs to a common reference year (2024 HK $). The manuscript have been extended with an explanation of this and a reference to the inflation index used.

**Compliance Assumptions:**

The base-case compliance rate of 52% appears low. Although sensitivity analyses include 75–100% compliance scenarios (S4 Appendix), the implications of low compliance on real-world effectiveness could be discussed more deeply. For example, could a biomarker-based approach also improve participation rates due to convenience?

Author response C-3:

We discuss in the manuscript that it is likely that a biomarker-based approach improve participation rates since this does not require ultrasound, hence becoming easier for the healthcare system to schedule and requiring less patient time for screening.

Our scenario analysis (Appendix S4) includes the following scenarios:

- Surveillance compliance rate: 100%
- Surveillance compliance rate: 75%
- Surveillance compliance rate: 75% for PIVKA-II-based screening and 52% for ultrasound-based screening.

In all scenarios, ‘PIVKA-II + AFP’ were preferred from a cost-effectiveness perspective

**Equity and Implementation Considerations:**

The discussion could briefly address potential barriers or enablers to adopting PIVKA-II + AFP at scale (e.g., laboratory capacity, reimbursement, clinician familiarity).

Including a brief cost–benefit or budget impact consideration would increase the paper’s practical policy value.

Author response C-4:

We have added a short paragraph to the “Discussion” that briefly addresses this.

Overall, the prospects of adopting ‘PIVKA-II + AFP’ into Hong Kong routine HCC surveillance are relatively good. AFP is already routinely utilised within HCC surveillance as part of the recommended standard of care (‘US + AFP’). Further, many Hong Kong hospitals already have access to the necessary infrastructure to adopt the technology, including the laboratory capacity and familiarity among clinicians.

**Source of Diagnostic Accuracy Data:**

Some key diagnostic accuracy parameters for PIVKA-II and PIVKA-II + AFP rely on “Roche Diagnostics, data on file.” For transparency and reproducibility, please describe the study design, patient population, and sample size of these unpublished data sources, or consider citing a peer-reviewed equivalent.

Author response C-5:

We agree with that ‘Data on file’ is insufficient as a reference for publication of the diagnostic accuracy data for ‘PIVKA-II + AFP’. Prompted by this comment, we attempted to investigate and describe the data further. However, following discussions with the data holder, we have concluded that we are unable to obtain and validate all study data within PLOS ONE’s required timeframe. However, we have been able to identify published alternative data on the diagnostic performance of ‘PIVKA-II + AFP’, and have decided to use this for the analyses instead of the performance data.

For non-cirrhotic patients, Nan et al. (2024) reported diagnostic performance for ‘PIVKA-II + AFP’ among Chinese patients with chronic hepatitis B. The similarities in patient characteristics between their population and the target population for this study makes this data suitable. Further, the data was reported by HCC stage, which enables the core analysis required for our cost-utility analysis; distinguishing between early-stage and later-stage performance is a requirement for properly assessing the impact of routine HCC surveillance. Compared to the data in our original analysis, Nan et al. (2024) reported somewhat lower diagnostic accuracy. This allows for a more conservative estimation, and the fact that our conclusions remain largely unchanged with this more conservative dataset increases our confidence in our findings.

For the diagnostic performance of ‘PIVKA-II + AFP’ among cirrhotic patients, this has previously been published by Pinjaroen et al. (2026). The methodology for obtaining this data was described by Garay et al. (2024). We have updated the references within the manuscript to reflect this.

**Minor Comments**

Abstract: In line 45 “cost-reduction of HK $7,296 per surveilled individual” with “cost saving of HK$7,296 per individual” for smoother readability.

Clarify that the analysis adopts a lifetime horizon from the payer perspective.

Methods: Plainly state the time horizon used (presumably lifetime) and justify the choice of discount rate (3%).

Define abbreviations (e.g., DCLC, OLT) upon first use in the main text.

Results: In Fig. 1 (model schematic), label “Death” as an absorbing state to make transitions clearer. you should explicitly mark or annotate the ‘Death’ node as absorbing. Or writing “Death (absorbing state)” in the diagram box itself.

Discussion: Consider referencing recent global cost-effectiveness evidence on HCC surveillance (e.g., 2023–2024 systematic reviews).

A concise paragraph contrasting findings with those from mainland China or Japan would situate the results in a broader regional context.

Language and Style: Minor grammatical corrections are needed (e.g., “dependance” in line 289→ “dependence”). Overall, the English language and structure are clear and professional.

References: Cite recent (2023–2024) global studies on HCC surveillance cost-effectiveness to contextualize findings.

Author response C-6:

We have addressed the reviewer’s feedback accordingly. The manuscript has been revised to improve the clarity of language, and all abbreviations have been defined upon first use in the main text. We have also clarified in the manuscript that the evaluation was conducted from a payer perspective using a lifetime horizon, together with the justification for applying a 3% discount rate. In addition, given that a previously cited cost‑effectiveness study from China supports our conclusions, we have extracted the relevant findings from that study to further contextualize and reinforce our observations. We note, however, that locally generated evidence on PIVKA‑II-based HCC surveillance remains limited.

### **Reviewer #3:**

The manuscript is very well written, and its clarity makes the findings readily accessible. The economic analysis suggests that surveillance of hepatocellular carcinoma (HCC) patients through the implementation of a protocol to induce and monitor PIVKA-II may represent a potentially economically dominant strategy under the assumptions modelled, and therefore merits further investigation. I would welcome the publication of these results.

However, the strength of these conclusions is undermined by the limited discussion of potential confounding factors (including disease severity, hepatic function, and concurrent therapies), as well as by the absence of consideration of the clinical consequences and risks associated with deliberately inducing PIVKA-II through modulation of the vitamin K pathway. In particular, the potential anticoagulant effects and associated bleeding risk—especially in patients with underlying liver dysfunction—warrant explicit discussion. Addressing these issues is essential to contextualise the economic findings and to support the clinical plausibility and safety of the proposed surveillance strategy.

Author response D-1:

We are grateful to hear that the reviewer considers our manuscript well written and worthy of publication.

The potential influence of confounding factors for the model’s underlying data is hard to rule out. Disease severity and hepatic function is discussed throughout the manuscript, including how liver cirrhosis affects the diagnostic performance of different screening methods. Since this is a modelling study based upon data published elsewhere, we cannot easily control for any concurrent therapies that patients in those studies may have received. However, our sensitivity and scenario analyses provides some confidence in the robustness of our conclusions, even if the underlying data would be updated.

We believe that the reviewer may have misunderstood the clinical consequences and risks associated with testing for PIVKA-II (Protein Induced by Vitamin K Absence or Antagonist-II; also known as des-gamma-carboxy prothrombin [DCP]). PIVKA-II is an abnormal form of prothrombin used as a biomarker. The patient’s blood serum is screened for the presence of this biomarker, which often indicates presence of HCC. PIVKA-II is not induced during testing. No harm befalls the patient during testing beyond the extraction of blood serum; this is a common procedure within healthcare and would be the same when testing patients for alpha-fetoprotein (AFP), the currently recommended standard-of-care in Hong Kong.

**6. PLOS authors have the option to publish the peer review history of their article (what does this mean?). If published, this will include your full peer review and any attached files.**

If you choose “no”, your identity will remain anonymous but your review may still be made public.

Do you want your identity to be public for this peer review? For information about this choice, including consent withdrawal, please see our Privacy Policy.

**Reviewer #1:** Yes: Mohsin Hassan Alvi

**Reviewer #2:** Yes: Montasir Ahmed

**Reviewer #3:** Yes: Francesco Bolstad

[NOTE: If reviewer comments were submitted as an attachment file, they will be attached to this email and accessible via the submission site. Please log into your account, locate the manuscript record, and check for the action link "View Attachments". If this link does not appear, there are no attachment files.]

## Further author instructions:

To ensure your figures meet our technical requirements, please review our figure guidelines: <https://journals.plos.org/plosone/s/figures>

You may also use PLOS’s free figure tool, NAAS, to help you prepare publication quality figures: <https://journals.plos.org/plosone/s/figures#loc-tools-for-figure-preparation>.

NAAS will assess whether your figures meet our technical requirements by comparing each figure against our figure specifications.

## References:

Garay OU, Ambühl LE, Bird TG, Barnes E, Irving WL, Walkley R, et al. Cost-Effectiveness of Hepatocellular Carcinoma Surveillance Strategies in Patients With Compensated Liver Cirrhosis in the United Kingdom. Value Health. 2024 Dec;27(12):1698–709

Nan Y, Garay OU, Lu X, Zhang Y, Xie L, Niu Z, Chen W. Early-stage hepatocellular carcinoma screening in patients with chronic hepatitis B in China: a cost-effectiveness analysis. J Comp Eff Res. 2024 Apr;13(4):e230146.

Nguyen et al. (2021) A Systematic Review and Narrative Synthesis of Health Economic Evaluations of Hepatocellular Carcinoma Screening Strategies. Value in Health, Volume 24, Issue 5, 733 – 743

Pinjaroen N, Pan-Ngum W, Poovorawan K, Wastlund D, Mueller F, Lu P, Yu RSS, Tangkijvanich P. Economic evaluation of biomarker-based surveillance for Hepatocellular Carcinoma in Thai patients with Compensated Liver Cirrhosis. PLoS One. 2026 Jan 5;21(1):e0337913. doi: 10.1371/journal.pone.0337913

Lui RNS, Mak LLY, Kung KN, et al. (2024) Protein induced by vitamin K absence-II for the surveillance and monitoring of hepatocellular carcinoma in Hong Kong. Hong Kong Medical Journal, 2024 Oct;30(5):418-421

Yau T, Tang VYF, Yao TJ, Fan ST, Lo CM, Poon RTP. Development of Hong Kong Liver Cancer staging system with treatment stratification for patients with hepatocellular carcinoma. Gastroenterology. 2014 Jun;146(7):1691-1700.e3.

Zhang M, Li Y, Fan Z, Shen D, Huang X, Yu Q, et al. Assessing health-related quality of life and health utilities in patients with chronic hepatitis B-related diseases in China: a cross-sectional study. 2021 Sep
