# Supplementary material for: Surveillance of hepatocellular carcinoma (HCC) patients using Protein Induced by Vitamin K (PIVKA-II): A cost-utility analysis for Hong Kong
Source: PLoS One. 2026 Jul 17;21(7):e0353882. doi: 10.1371/journal.pone.0353882 (PMC13378965; doi:10.1371/journal.pone.0353882)
Supplement: Attachment — Submitted filename: Cost-utility analysis in Hong Kong.docx [file pone.0353882.s005.docx]

**Overall Assessment**

This manuscript presents a well-structured and timely cost-utility analysis evaluating the use of PIVKA-II combined with AFP for HCC surveillance in Hong Kong. The authors adapt a Markov microsimulation model to local epidemiological and economic conditions, providing valuable insight into the potential role of biomarker-based surveillance in resource-limited settings. The study addresses an important gap in liver cancer screening policy and is highly relevant to clinical and public health decision-making in regions with high HBV prevalence.

The manuscript is generally well written, clearly structured, and appropriately referenced. The modelling framework, assumptions, and results are presented with transparency, and the conclusions are well aligned with the findings. Some of the major strengths of this manuscript have relevance and policy impact, methodological Transparency, comprehensive sensitivity analysis and balanced discussion. However, some clarifications and improvements would enhance the scientific rigour and reproducibility of the study.

**Major Comments**

**Model Validation and Calibration:**

Although the model is adapted from prior work, it would be helpful to describe how the model was validated or calibrated against observed Hong Kong data (e.g., incidence, stage distribution, or survival outcomes).

A visual comparison between simulated and empirical HCC detection rates or survival curves could strengthen confidence in model accuracy.

**Perspective and Cost Components:**

The analysis adopts a healthcare system (payer) perspective. However, some key cost categories such as patient time costs or indirect costs are not discussed. Clarifying whether these were intentionally excluded and their potential influence on results would be valuable.

Please specify whether costs are adjusted to a reference year (e.g., 2024 HK$) and describe the inflation or exchange rate method used.

**Compliance Assumptions:**

The base-case compliance rate of 52% appears low. Although sensitivity analyses include 75–100% compliance scenarios (S4 Appendix), the implications of low compliance on real-world effectiveness could be discussed more deeply. For example, could a biomarker-based approach also improve participation rates due to convenience?

**Equity and Implementation Considerations:**

The discussion could briefly address potential barriers or enablers to adopting PIVKA-II + AFP at scale (e.g., laboratory capacity, reimbursement, clinician familiarity).

Including a brief cost–benefit or budget impact consideration would increase the paper’s practical policy value.

**Source of Diagnostic Accuracy Data:**

Some key diagnostic accuracy parameters for PIVKA-II and PIVKA-II + AFP rely on “Roche Diagnostics, data on file.” For transparency and reproducibility, please describe the study design, patient population, and sample size of these unpublished data sources, or consider citing a peer-reviewed equivalent.

**Minor Comments**

**Abstract:** In line 45 “cost-reduction of HK $7,296 per surveilled individual” with “cost saving of HK$7,296 per individual” for smoother readability.

Clarify that the analysis adopts a *lifetime horizon* from the payer perspective.

**Methods:** Plainly state the time horizon used (presumably lifetime) and justify the choice of discount rate (3%).

Define abbreviations (e.g., DCLC, OLT) upon first use in the main text.

**Results:** In Fig. 1 (model schematic), label “Death” as an absorbing state to make transitions clearer. you should explicitly mark or annotate the **‘Death’** node as *absorbing*. Or writing “Death (absorbing state)” in the diagram box itself.

**Discussion:** Consider referencing recent global cost-effectiveness evidence on HCC surveillance (e.g., 2023–2024 systematic reviews).

A concise paragraph contrasting findings with those from mainland China or Japan would situate the results in a broader regional context.

**Language and Style:** Minor grammatical corrections are needed (e.g., “dependance” in line 289→ “dependence”). Overall, the English language and structure are clear and professional.

**References:** Cite recent (2023–2024) global studies on HCC surveillance cost-effectiveness to contextualise findings.
