# Supplementary material for: Surveillance of hepatocellular carcinoma (HCC) patients using Protein Induced by Vitamin K (PIVKA-II): A cost-utility analysis for Hong Kong
Source: PLoS One. 2026 Jul 17;21(7):e0353882. doi: 10.1371/journal.pone.0353882 (PMC13378965; doi:10.1371/journal.pone.0353882)
Supplement: Peer review history

# Peer review: Decision Letter 0

**DOI:** 10.1371/journal.pone.0353882.r001

**Authors:** Xinjun Lu

4 Jan 2026

PONE-D-25-51132

Surveillance of hepatocellular carcinoma (HCC) patients using Protein Induced by Vitamin K (PIVKA-II): A cost-utility analysis for Hong Kong

PLOS One

Dear Dr. Wastlund,

Thank you for submitting your manuscript to PLOS ONE. After careful consideration, we feel that it has merit but does not fully meet PLOS ONE’s publication criteria as it currently stands. Therefore, we invite you to submit a revised version of the manuscript that addresses the points raised during the review process.

Upon internal evaluation of the reviews provided, we kindly request you to disregard the reviewer report provided by Reviewer 1. No amendments are required in response to reviewer 1’s comments

Please submit your revised manuscript by Feb 18 2026 11:59PM. If you will need more time than this to complete your revisions, please reply to this message or contact the journal office at plosone@plos.org. When you're ready to submit your revision, log on to https://www.editorialmanager.com/pone/ and select the 'Submissions Needing Revision' folder to locate your manuscript file.

Please include the following items when submitting your revised manuscript:

- A letter that responds to each point raised by the academic editor and reviewer(s). You should upload this letter as a separate file labeled 'Response to Reviewers'.
- A marked-up copy of your manuscript that highlights changes made to the original version. You should upload this as a separate file labeled 'Revised Manuscript with Track Changes'.
- An unmarked version of your revised paper without tracked changes. You should upload this as a separate file labeled 'Manuscript'.

If you would like to make changes to your financial disclosure, please include your updated statement in your cover letter. Guidelines for resubmitting your figure files are available below the reviewer comments at the end of this letter.

If applicable, we recommend that you deposit your laboratory protocols in protocols.io to enhance the reproducibility of your results. Protocols.io assigns your protocol its own identifier (DOI) so that it can be cited independently in the future. For instructions see: https://journals.plos.org/plosone/s/submission-guidelines#loc-laboratory-protocols. Additionally, PLOS ONE offers an option for publishing peer-reviewed Lab Protocol articles, which describe protocols hosted on protocols.io. Read more information on sharing protocols at https://plos.org/protocols?utm_medium=editorial-email&utm_source=authorletters&utm_campaign=protocols.

We look forward to receiving your revised manuscript.

Kind regards,

Xinjun Lu

Academic Editor

PLOS One

Journal Requirements:

When submitting your revision, we need you to address these additional requirements.

1. Please ensure that your manuscript meets PLOS ONE's style requirements, including those for file naming. The PLOS ONE style templates can be found at

https://journals.plos.org/plosone/s/file?id=wjVg/PLOSOne_formatting_sample_main_body.pdf and

https://journals.plos.org/plosone/s/file?id=ba62/PLOSOne_formatting_sample_title_authors_affiliations.pdf

2. Please note that PLOS One has specific guidelines on code sharing for submissions in which author-generated code underpins the findings in the manuscript. In these cases, we expect all author-generated code to be made available without restrictions upon publication of the work. Please review our guidelines at https://journals.plos.org/plosone/s/materials-and-software-sharing#loc-sharing-code and ensure that your code is shared in a way that follows best practice and facilitates reproducibility and reuse.

3. Please update your submission to use the PLOS LaTeX template. The template and more information on our requirements for LaTeX submissions can be found at http://journals.plos.org/plosone/s/latex

4. We note that the grant information you provided in the ‘Funding Information’ and ‘Financial Disclosure’ sections do not match.

When you resubmit, please ensure that you provide the correct grant numbers for the awards you received for your study in the ‘Funding Information’ section.

5. Thank you for stating the following financial disclosure:

“The health economic model used for this analysis was funded by Roche Diagnostics Asia Pacific Ltd, which also provided access to data on diagnostic accuracy. QZ and JC are previous employees of Roche Diagnostics. DW and TT are consultants at Vista Health, a company that have received funding from Roche Diagnostics for evaluating the cost-effectiveness of alternative HCC surveillance methods. MFY has received compensation at fair market value for his consultancy in model advisory boards of Roche Diagnostics and for delivering lectures organised by Roche Diagnostics. The opinions expressed in this publication are those of the authors and they do not necessarily reflect the view of the funder.”

Please state what role the funders took in the study.  If the funders had no role, please state: "The funders had no role in study design, data collection and analysis, decision to publish, or preparation of the manuscript."

If this statement is not correct you must amend it as needed.

Please include this amended Role of Funder statement in your cover letter; we will change the online submission form on your behalf.

6. Thank you for stating the following financial disclosure:

“The health economic model used for this analysis was funded by Roche Diagnostics Asia Pacific Ltd, which also provided access to data on diagnostic accuracy. QZ and JC are previous employees of Roche Diagnostics. DW and TT are consultants at Vista Health, a company that have received funding from Roche Diagnostics for evaluating the cost-effectiveness of alternative HCC surveillance methods. MFY has received compensation at fair market value for his consultancy in model advisory boards of Roche Diagnostics and for delivering lectures organised by Roche Diagnostics. The opinions expressed in this publication are those of the authors and they do not necessarily reflect the view of the funder.”

We note that one or more of the authors is affiliated with the funding organization, indicating the funder may have had some role in the design, data collection, analysis or preparation of your manuscript for publication; in other words, the funder played an indirect role through the participation of the co-authors. If the funding organization did not play a role in the study design, data collection and analysis, decision to publish, or preparation of the manuscript and only provided financial support in the form of authors' salaries and/or research materials, please do the following:

1. Review your statements relating to the author contributions, and ensure you have specifically and accurately indicated the role(s) that these authors had in your study. These amendments should be made in the online form.

2. Confirm in your cover letter that you agree with the following statement, and we will change the online submission form on your behalf:

“The funder provided support in the form of salaries for authors [insert relevant initials], but did not have any additional role in the study design, data collection and analysis, decision to publish, or preparation of the manuscript. The specific roles of these authors are articulated in the ‘author contributions’ section.

7. In the online submission form, you indicated that “All data used for this analysis are included in the article. In most cases, data have been obtained from other published studies, and these are referenced within the manuscript.

All supporting calculations and analyses can be made available upon reasonable request to the corresponding author. “

All PLOS journals now require all data underlying the findings described in their manuscript to be freely available to other researchers, either 1. In a public repository, 2. Within the manuscript itself, or 3. Uploaded as supplementary information.

This policy applies to all data except where public deposition would breach compliance with the protocol approved by your research ethics board. If your data cannot be made publicly available for ethical or legal reasons (e.g., public availability would compromise patient privacy), please explain your reasons on resubmission and your exemption request will be escalated for approval.

8. If the reviewer comments include a recommendation to cite specific previously published works, please review and evaluate these publications to determine whether they are relevant and should be cited. There is no requirement to cite these works unless the editor has indicated otherwise.

[Note: HTML markup is below. Please do not edit.]

Reviewers' comments:

Reviewer's Responses to Questions

**Comments to the Author**

1. Is the manuscript technically sound, and do the data support the conclusions?

The manuscript must describe a technically sound piece of scientific research with data that supports the conclusions. Experiments must have been conducted rigorously, with appropriate controls, replication, and sample sizes. The conclusions must be drawn appropriately based on the data presented.

Reviewer #1: Yes

Reviewer #2: Yes

Reviewer #3: Partly

**********

2. Has the statistical analysis been performed appropriately and rigorously?

Reviewer #1: Yes

Reviewer #2: Yes

Reviewer #3: Yes

**********

3. Have the authors made all data underlying the findings in their manuscript fully available?

The PLOS Data policy requires authors to make all data underlying the findings described in their manuscript fully available without restriction, with rare exception (please refer to the Data Availability Statement in the manuscript PDF file). The data should be provided as part of the manuscript or its supporting information, or deposited to a public repository. For example, in addition to summary statistics, the data points behind means, medians and variance measures should be available. If there are restrictions on publicly sharing data—e.g. participant privacy or use of data from a third party—those must be specified.

Reviewer #1: Yes

Reviewer #2: Yes

Reviewer #3: Yes

**********

4. Is the manuscript presented in an intelligible fashion and written in standard English?

PLOS ONE does not copyedit accepted manuscripts, so the language in submitted articles must be clear, correct, and unambiguous. Any typographical or grammatical errors should be corrected at revision, so please note any specific errors here.

Reviewer #1: Yes

Reviewer #2: Yes

Reviewer #3: Yes

**********

5. Review Comments to the Author

Please use the space provided to explain your answers to the questions above. You may also include additional comments for the author, including concerns about dual publication, research ethics, or publication ethics. (Please upload your review as an attachment if it exceeds 20,000 characters)

Reviewer #1: The manuscript is well written, clearly structured, and addresses an important and timely topic in hepatocellular carcinoma (HCC) surveillance. The authors present a cost-utility analysis comparing “PIVKA-II + AFP” with the current standard of care “US + AFP” in Hong Kong, using a Markov microsimulation model.

The study is relevant, methodologically sound, and offers valuable insights into health economic decision-making and screening policy in a high-burden region. Overall, it provides a meaningful contribution to the field. However, a number of points should be considered to further strengthen the manuscript.

• The abstract is informative, but it could briefly clarify that the study uses a model-based economic evaluation relying on published data rather than primary patient-level data.

• The introduction provides good background but would benefit from a clearer statement of novelty—what this study adds beyond previous cost-effectiveness studies of biomarker-based HCC surveillance in Asia.

• The rationale for focusing on Hong Kong is well justified; however, the authors could discuss the potential generalizability of their findings to other similar health systems or regions with limited ultrasound capacity.

• The Methods section is comprehensive, but additional details on model validation, calibration, and verification by local experts would improve transparency. It would be useful to mention whether the model was externally validated or cross-checked with real-world data.

• The choice of the Markov microsimulation model is appropriate, yet a schematic justification for the health states and transition logic (e.g., CHB → LC → HCC stages) should be presented in clearer terms for readers unfamiliar with economic modeling.

• The derivation of QALYs should be described in greater detail, including how utility values were obtained, whether local Hong Kong data were available, and how these values were applied across disease states.

• Transition probabilities and epidemiological parameters appear to be drawn from a mixture of local and international sources; the paper should clarify how these were harmonized or adapted to Hong Kong’s context.

• The time horizon and discount rate (3%) are standard; however, the results would be more robust if sensitivity analyses around these parameters were presented explicitly.

• Cost inputs from older studies (e.g., Yuen et al. 2000) should either be updated or clearly adjusted for inflation to reflect current prices. The costing year should be explicitly stated.

• The analysis correctly adopts a health system payer perspective, but the exclusion of indirect or societal costs (e.g., productivity loss) should be noted and justified.

• The incremental cost-effectiveness ratio (ICER) results are well calculated, but the authors could improve presentation by including the base-case ICERs and the variation observed across sensitivity scenarios directly in the Results section.

• A cost-effectiveness acceptability curve (CEAC) or scatter plot from the probabilistic sensitivity analysis would help readers visually assess the uncertainty around cost-effectiveness.

• The chosen cost-effectiveness threshold (GDP per capita of HK$422,242 per QALY) is reasonable, but the discussion should acknowledge that there is no official threshold in Hong Kong and explore how results might differ under alternative thresholds.

• The financial disclosure statement indicates strong involvement of Roche Diagnostics, including data provision and consultancy relationships. This potential source of bias should be discussed more openly within the limitations section.

• Some diagnostic accuracy data are listed as “Roche diagnostics, data on file.” The authors should clarify whether these values have been independently validated or published in peer-reviewed form.

• The Results section is detailed and logically organized, but Table 2 could be simplified or moved in part to supplementary materials to improve readability.

• Subgroup and sensitivity analyses are well conceived, though a clearer explanation of the drivers of cost-effectiveness between cirrhotic and non-cirrhotic groups would help interpretation.

• The discussion effectively summarizes the main findings but should include a stronger acknowledgment that model-based results depend on assumptions and are not a substitute for real-world clinical evidence.

• The policy implications could be highlighted more strongly—specifically, how biomarker-based surveillance might complement, rather than replace, ultrasound within Hong Kong’s HCC screening framework.

• The conclusion could be shortened and focused more on the practical implications rather than restating quantitative findings already presented in the Results section.

• The limitations section should emphasize potential uncertainty due to parameter estimates derived from literature and manufacturer data, and discuss the absence of head-to-head comparative trials.

• The ethical statement reads “N/A.” The authors may clarify why ethical approval was not applicable, given that secondary data sources were used.

• Figures and tables should use consistent decimal places, abbreviations, and units. Minor language edits would further improve readability and flow.

• The data availability statement should ensure compliance with PLOS ONE’s open-data policy by specifying where model inputs and simulation codes can be accessed.

• Future studies could explore real-world comparative data between “PIVKA-II + AFP” and “US + AFP,” potentially including combinations with ultrasound or other biomarkers to confirm the model’s assumptions.

Reviewer #2: Overall Assessment

This manuscript presents a well-structured and timely cost-utility analysis evaluating the use of PIVKA-II combined with AFP for HCC surveillance in Hong Kong. The authors adapt a Markov microsimulation model to local epidemiological and economic conditions, providing valuable insight into the potential role of biomarker-based surveillance in resource-limited settings. The study addresses an important gap in liver cancer screening policy and is highly relevant to clinical and public health decision-making in regions with high HBV prevalence.

The manuscript is generally well written, clearly structured, and appropriately referenced. The modelling framework, assumptions, and results are presented with transparency, and the conclusions are well aligned with the findings. Some of the major strengths of this manuscript have relevance and policy impact, methodological Transparency, comprehensive sensitivity analysis and balanced discussion. However, some clarifications and improvements would enhance the scientific rigour and reproducibility of the study.

Major Comments

Model Validation and Calibration:

Although the model is adapted from prior work, it would be helpful to describe how the model was validated or calibrated against observed Hong Kong data (e.g., incidence, stage distribution, or survival outcomes).

A visual comparison between simulated and empirical HCC detection rates or survival curves could strengthen confidence in model accuracy.

Perspective and Cost Components:

The analysis adopts a healthcare system (payer) perspective. However, some key cost categories such as patient time costs or indirect costs are not discussed. Clarifying whether these were intentionally excluded and their potential influence on results would be valuable.

Please specify whether costs are adjusted to a reference year (e.g., 2024 HK$) and describe the inflation or exchange rate method used.

Compliance Assumptions:

The base-case compliance rate of 52% appears low. Although sensitivity analyses include 75–100% compliance scenarios (S4 Appendix), the implications of low compliance on real-world effectiveness could be discussed more deeply. For example, could a biomarker-based approach also improve participation rates due to convenience?

Equity and Implementation Considerations:

The discussion could briefly address potential barriers or enablers to adopting PIVKA-II + AFP at scale (e.g., laboratory capacity, reimbursement, clinician familiarity).

Including a brief cost–benefit or budget impact consideration would increase the paper’s practical policy value.

Source of Diagnostic Accuracy Data:

Some key diagnostic accuracy parameters for PIVKA-II and PIVKA-II + AFP rely on “Roche Diagnostics, data on file.” For transparency and reproducibility, please describe the study design, patient population, and sample size of these unpublished data sources, or consider citing a peer-reviewed equivalent.

Minor Comments

Abstract: In line 45 “cost-reduction of HK $7,296 per surveilled individual” with “cost saving of HK$7,296 per individual” for smoother readability.

Clarify that the analysis adopts a lifetime horizon from the payer perspective.

Methods: Plainly state the time horizon used (presumably lifetime) and justify the choice of discount rate (3%).

Define abbreviations (e.g., DCLC, OLT) upon first use in the main text.

Results: In Fig. 1 (model schematic), label “Death” as an absorbing state to make transitions clearer. you should explicitly mark or annotate the ‘Death’ node as absorbing. Or writing “Death (absorbing state)” in the diagram box itself.

Discussion: Consider referencing recent global cost-effectiveness evidence on HCC surveillance (e.g., 2023–2024 systematic reviews).

A concise paragraph contrasting findings with those from mainland China or Japan would situate the results in a broader regional context.

Language and Style: Minor grammatical corrections are needed (e.g., “dependance” in line 289→ “dependence”). Overall, the English language and structure are clear and professional.

References: Cite recent (2023–2024) global studies on HCC surveillance cost-effectiveness to contextualize findings.

Reviewer #3: The manuscript is very well written, and its clarity makes the findings readily accessible. The economic analysis suggests that surveillance of hepatocellular carcinoma (HCC) patients through the implementation of a protocol to induce and monitor PIVKA-II may represent a potentially economically dominant strategy under the assumptions modelled, and therefore merits further investigation. I would welcome the publication of these results.

However, the strength of these conclusions is undermined by the limited discussion of potential confounding factors (including disease severity, hepatic function, and concurrent therapies), as well as by the absence of consideration of the clinical consequences and risks associated with deliberately inducing PIVKA-II through modulation of the vitamin K pathway. In particular, the potential anticoagulant effects and associated bleeding risk—especially in patients with underlying liver dysfunction—warrant explicit discussion. Addressing these issues is essential to contextualise the economic findings and to support the clinical plausibility and safety of the proposed surveillance strategy.

**********

6. PLOS authors have the option to publish the peer review history of their article (what does this mean?). If published, this will include your full peer review and any attached files.

If you choose “no”, your identity will remain anonymous but your review may still be made public.

**Do you want your identity to be public for this peer review?**  For information about this choice, including consent withdrawal, please see our Privacy Policy.

Reviewer #1: **Yes:** Mohsin Hassan Alvi

Reviewer #2: **Yes:** Montasir Ahmed

Reviewer #3: **Yes:** Francesco Bolstad

**********

[NOTE: If reviewer comments were submitted as an attachment file, they will be attached to this email and accessible via the submission site. Please log into your account, locate the manuscript record, and check for the action link "View Attachments". If this link does not appear, there are no attachment files.]

To ensure your figures meet our technical requirements, please review our figure guidelines: https://journals.plos.org/plosone/s/figures

You may also use PLOS’s free figure tool, NAAS, to help you prepare publication quality figures: https://journals.plos.org/plosone/s/figures#loc-tools-for-figure-preparation.

NAAS will assess whether your figures meet our technical requirements by comparing each figure against our figure specifications.

Attachment Submitted filename: Cost-utility analysis in Hong Kong.docx

# Author comment: Author response to Decision Letter 1

**DOI:** 10.1371/journal.pone.0353882.r002

31 Mar 2026

We have provided all our responses to reviewer and editor comments in a submitted document: "Response to Reviewers v3.0 20260331"

Attachment Submitted filename: Response to Reviewers v3.0 20260331.docx

# Peer review: Decision Letter 1

**DOI:** 10.1371/journal.pone.0353882.r003

**Authors:** Xinjun Lu, Jee-Fu Huang

30 Jun 2026

Surveillance of hepatocellular carcinoma (HCC) patients using Protein Induced by Vitamin K (PIVKA-II): A cost-utility analysis for Hong Kong

PONE-D-25-51132R1

Dear Dr. Wastlund,

We’re pleased to inform you that your manuscript has been judged scientifically suitable for publication and will be formally accepted for publication once it meets all outstanding technical requirements.

Within one week, you’ll receive an e-mail detailing the required amendments. When these have been addressed, you’ll receive a formal acceptance letter and your manuscript will be scheduled for publication.

An invoice will be generated when your article is formally accepted. Please note, if your institution has a publishing partnership with PLOS and your article meets the relevant criteria, all or part of your publication costs will be covered. Please make sure your user information is up-to-date by logging into Editorial Manager at Editorial Manager® and clicking the ‘Update My Information' link at the top of the page. For questions related to billing, please contact billing support.

If your institution or institutions have a press office, please notify them about your upcoming paper to help maximize its impact. If they’ll be preparing press materials, please inform our press team as soon as possible -- no later than 48 hours after receiving the formal acceptance. Your manuscript will remain under strict press embargo until 2 pm Eastern Time on the date of publication. For more information, please contact onepress@plos.org.

Kind regards,

Jee-Fu Huang, M.D., Ph.D.

Academic Editor

PLOS One

Additional Editor Comments (optional):

Reviewers' comments:

Reviewer's Responses to Questions

**Comments to the Author**

1. If the authors have adequately addressed your comments raised in a previous round of review and you feel that this manuscript is now acceptable for publication, you may indicate that here to bypass the “Comments to the Author” section, enter your conflict of interest statement in the “Confidential to Editor” section, and submit your "Accept" recommendation.

Reviewer #3: All comments have been addressed

Reviewer #4: All comments have been addressed

**********

2. Is the manuscript technically sound, and do the data support the conclusions?

The manuscript must describe a technically sound piece of scientific research with data that supports the conclusions. Experiments must have been conducted rigorously, with appropriate controls, replication, and sample sizes. The conclusions must be drawn appropriately based on the data presented.

Reviewer #3: Yes

Reviewer #4: Yes

**********

3. Has the statistical analysis been performed appropriately and rigorously?

Reviewer #3: Yes

Reviewer #4: Yes

**********

4. Have the authors made all data underlying the findings in their manuscript fully available?

The PLOS Data policy requires authors to make all data underlying the findings described in their manuscript fully available without restriction, with rare exception (please refer to the Data Availability Statement in the manuscript PDF file). The data should be provided as part of the manuscript or its supporting information, or deposited to a public repository. For example, in addition to summary statistics, the data points behind means, medians and variance measures should be available. If there are restrictions on publicly sharing data—e.g. participant privacy or use of data from a third party—those must be specified.

Reviewer #3: Yes

Reviewer #4: Yes

**********

5. Is the manuscript presented in an intelligible fashion and written in standard English?

PLOS ONE does not copyedit accepted manuscripts, so the language in submitted articles must be clear, correct, and unambiguous. Any typographical or grammatical errors should be corrected at revision, so please note any specific errors here.

Reviewer #3: Yes

Reviewer #4: Yes

**********

6. Review Comments to the Author

Please use the space provided to explain your answers to the questions above. You may also include additional comments for the author, including concerns about dual publication, research ethics, or publication ethics. (Please upload your review as an attachment if it exceeds 20,000 characters)

Reviewer #3: I thank the authors for their detailed response and for clarifying the nature of the PIVKA-II biomarker. I acknowledge that my previous concerns regarding the deliberate induction of PIVKA-II and associated anticoagulant risks were based on a misunderstanding of the diagnostic protocol. I am satisfied with the explanation that the surveillance strategy involves a standard non-invasive serum assay with a safety profile identical to current standard-of-care biomarkers.

Furthermore, I appreciate the inclusion of updated diagnostic accuracy data (e.g., Pinjaroen et al., 2026) and the further stratification of the model by disease severity (CHB vs. LC). These additions sufficiently address my previous concerns regarding confounding factors and support the clinical plausibility of the economic findings. I have no further concerns and recommend the manuscript for publication.

Reviewer #4: Authors addressed raised issues appropriately. So, this is enough to be accepted and published in the PLos ONe.

**********

7. PLOS authors have the option to publish the peer review history of their article (what does this mean?). If published, this will include your full peer review and any attached files.

If you choose “no”, your identity will remain anonymous but your review may still be made public.

**Do you want your identity to be public for this peer review?**  For information about this choice, including consent withdrawal, please see our Privacy Policy.

Reviewer #3: **Yes:** Francesco Bolstad

Reviewer #4: No

**********

# Editor report: Acceptance letter

**DOI:** 10.1371/journal.pone.0353882.r004

**Authors:** Xinjun Lu, Jee-Fu Huang

PONE-D-25-51132R1

PLOS One

Dear Dr. Wastlund,

I'm pleased to inform you that your manuscript has been deemed suitable for publication in PLOS One. Congratulations! Your manuscript is now being handed over to our production team.

At this stage, our production department will prepare your paper for publication. This includes ensuring the following:

* All references, tables, and figures are properly cited

* All relevant supporting information is included in the manuscript submission,

* There are no issues that prevent the paper from being properly typeset

You will receive further instructions from the production team, including instructions on how to review your proof when it is ready. Please keep in mind that we are working through a large volume of accepted articles, so please give us a few days to review your paper and let you know the next and final steps.

Lastly, if your institution or institutions have a press office, please let them know about your upcoming paper now to help maximize its impact. If they'll be preparing press materials, please inform our press team within the next 48 hours. Your manuscript will remain under strict press embargo until 2 pm Eastern Time on the date of publication. For more information, please contact onepress@plos.org.

You will receive an invoice from PLOS for your publication fee after your manuscript has reached the completed accept phase. If you receive an email requesting payment before acceptance or for any other service, this may be a phishing scheme. Learn how to identify phishing emails and protect your accounts at https://explore.plos.org/phishing.

If we can help with anything else, please email us at customercare@plos.org.

Thank you for submitting your work to PLOS ONE and supporting open access.

Kind regards,

PLOS ONE Editorial Office Staff

on behalf of

Dr. Jee-Fu Huang

Academic Editor

PLOS One
